# The selective post-translational processing of transcription factor Nrf1 yields distinct isoforms that dictate its ability to differentially regulate gene expression

**DOI:** 10.1038/srep12983

**Published:** 2015-08-13

**Authors:** Yiguo Zhang, Shaojun Li, Yuancai Xiang, Lu Qiu, Huakan Zhao, John D. Hayes

**Affiliations:** 1 The NSFC-funded Laboratory of Cell Biochemistry and Gene Regulation, College of Medical Bioengineering and Faculty of Life Sciences, Chongqing University, No. 174 Shazheng Street, Shapingba District, Chongqing 400044, China; 2 Jacqui Wood Cancer Centre, James Arrott Drive, Division of Cancer Research, Medical Research Institute, Ninewells Hospital & Medical School, University of Dundee, DD1 9SY, Scotland, UK

## Abstract

Upon translation, the N-terminal homology box 1 (NHB1) signal anchor sequence of Nrf1 integrates it within the endoplasmic reticulum (ER) whilst its transactivation domains [TADs, including acidic domain 1 (AD1), the flanking Asn/Ser/Thr-rich (NST) domain and AD2] are transiently translocated into the ER lumen, whereupon the NST domain is glycosylated to yield an inactive 120-kDa glycoprotein. Subsequently, these TADs are retrotranslocated into extra-luminal subcellular compartments, where Nrf1 is deglycosylated to yield an active 95-kDa isoform. Herein, we report that AD1 and AD2 are required for the stability of the 120-kDa Nrf1 glycoprotein, but not that of the non-glycosylated/de-glycosylated 95-kDa isoform. Degrons within AD1 do not promote proteolytic degradation of the 120-kDa Nrf1 glycoprotein. However, repositioning of AD2-adjoining degrons (i.e. DSGLS-containing SDS1 and PEST2 sequences) into the cyto/nucleoplasm enables selective topovectorial processing of Nrf1 by the proteasome and/or calpains to generate a cleaved active 85-kDa Nrf1 or a dominant-negative 36-kDa Nrf1γ. Production of Nrf1γ is abolished by removal of SDS1 or PEST2 degrons, whereas production of the cleaved 85-kDa Nrf1 is blocked by deletion of the ER luminal-anchoring NHB2 sequence (aa 81–106). Importantly, Nrf1 activity is positively and/or negatively regulated by distinct doses of proteasome and calpain inhibitors.

The cap’n’collar (CNC) basic-region leucine zipper (bZIP) family of transcription factors includes the *Drosophila* Cnc protein, the vertebrate activator nuclear factor-erythroid 2 (NF-E2) p45 and its related factors Nrf1 (including its long form TCF11 and its short form LCR-F1/Nrf1β), Nrf2 and Nrf3, as well as the transcription repressors Bach1 and Bach2 (refs 1, 2, 3, 4, 5). The CNC-bZIP proteins heterodimerize with small Maf or other bZIP proteins before they bind to antioxidant/electrophile response element (ARE/EpRE) sequences in their target gene promoters. This family of transcription factors controls critical homeostatic and developmental pathways because they regulate both basal and inducible expression of ARE-battery genes, which encode antioxidant proteins, detoxification enzymes, metabolic enzymes and 26S proteosomal subunits. In mammals, Nrf1 and Nrf2 are ubiquitously expressed and represent the principal CNC-bZIP factors that regulate ARE-driven genes in non-hematological tissues^6^,^7^,^8^.

Most research into CNC-bZIP proteins has focused on Nrf2, which is a master regulator of adaptive responses to oxidative stressors and electrophiles^9^,^10^,^11^. However, Nrf2 is not essential for development because global knockout of its gene in mice yields viable animals^12^, and whilst *Nrf2*^−/−^ mice do not spontaneously develop cancer, they are more susceptible than wild-type mice to carcinogens^13^. By contrast with Nrf2, which is a soluble protein, relatively less is known about the membrane-bound Nrf1 factor, although global knockout of its gene in the mouse causes embryonic lethality and severe oxidative stress^14^,^15^,^16^,^17^. Moreover, conditional knockout of *Nrf1* in mouse liver, brain and bone results in non-alcoholic steatohepatitis and hepatoma^18^,^19^, neurodegeneration^20^,^21^, and reduced bone size^22^. The fact that Nrf1 is essential for maintaining cellular homeostasis and organ integrity, demonstrates that it fulfils a unique and indispensable function(s).

Nrf1 is an integral membrane protein that is targeted to the endoplasmic reticulum (ER) through its N-terminal homology box 1 (NHB1, aa 11–30) sequence, which lacks a signal peptidase (SPase) cleavage site^23^,^24^. Importantly, the SPase-uncleavable NHB1 peptide defines the topology of integration of Nrf1 within and around ER membranes^25^,^26^. Thus by mechanisms that are not understood, the NHB1 sequence and adjoining regions determine whether Nrf1 is either retained in the ER or sorted out into the nuclear envelope membrane^8^,^23^. Our previous work revealed that the overall membrane-topology of Nrf1 is determined by the NHB1-associated transmembrane-1 (TM1, aa 7–24) region, in cooperation with other semihydrophobic amphipathic segments (e.g. TMi, TMp and TMc), but it is evidently distinct from those of the classic membrane-associated transcription factors ATF6 and SREBP1 (refs 24,26,27). Within the N-terminal domain (NTD, aa 1–124) of Nrf1, the NHB2 (aa 81–106) sequence serves as a topological anchor on the luminal side of the ER membrane^28^. It is notable that the NHB2-adjoining peptides do not possess Site-1 or Site-2 protease-mediated proteolytic cleavage sites, such as those in ATF6 and SREBP1 (refs 24,26).

During co-translational topogenesis, Nrf1 is anchored within ER membranes through its TM1 region and, subsequently, its acidic transactivation domain (TAD) sequences are transiently translocated into the ER lumen, where the Asn/Ser/Thr-rich (NST) domain, situated between acidic domain 1 (AD1) and AD2 (see Fig. S1), is glycosylated in the presence of glucose to yield an inactive 120-kDa Nrf1 glycoprotein^25^,^26^. Under appropriate stimuli, the TAD sequences are dynamically repartitioned out of the ER and retrotranslocated across membranes into the cyto/nucleoplasmic subcellular compartments, where the NST glycodomain of Nrf1 is deglycosylated to generate an active 95-kDa protein. During this vectorial process, the repositioning of potential TAD-adjoining degrons in Nrf1 [i.e. the Cdc4 phosphodegron (CPD), the β-TrCP-binding DSGLS motif, and the PEST sequences^29^,^30^,^31^] from within the ER lumen to the cyto/nucleoplasmic side allows selective proteolytic processing to yield multiple Nrf1 isoforms of between 85-kDa and 25-kDa, which together control its transcription activity^25^,^26^. However, the process by which the selective proteolysis of Nrf1 is controlled has not been elucidated.

In order to provide a better understanding of the molecular basis by which post-translational processing of Nrf1 generates distinct isoforms, we have herein examined whether: (i) the topological repartitioning of Nrf1 from the ER lumen into the cyto/nucleoplasmic side of membranes enables it to be proteolytically processed by cytosolic proteases; (ii) the vectorial processing of Nrf1 is affected by NHB2; (iii) the putative TAD-adjoining degrons contribute to the selective proteolytic processing of Nrf1; (iv) the proteolytic processing of Nrf1 by the proteasome and/or calpain regulates its transcription activity to differentially mediate the expression of ARE-driven genes; and (v) a bidirectional regulatory feedback circuit exists between Nrf1 and the proteasome.

## Results

### Dynamic movement of Nrf1 from the ER luminal side of membranes into the cytoplasmic side renders it susceptible to protease attack

**Figure 1:**
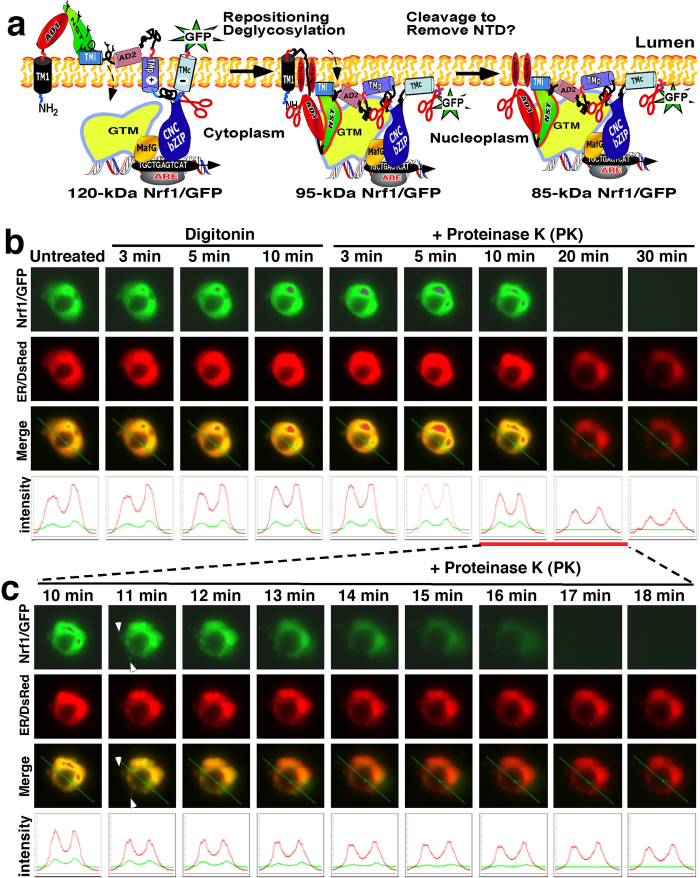
Live-cell imaging of Nrf1/GFP that dynamically moves out of the ER into the cytoplasm. (**a**) Distinct membrane-topologies of 120-kDa Nrf1/GFP, 95-kDa Nrf1/GFP and 85-kDa Nrf1/GFP around and within the ER are schematically shown. Nrf1 has been fused C-terminally by GFP, which faces the luminal side of membranes during the initial topogenesis. Since Nrf1 is a mobile membrane-protein that entails dynamic topologies, the overall membrane-topological folding of glycosylated 120-kDa Nrf1/GFP is distinct from that of non-glycosylated/de-glyosylated 95-kDa Nrf1/GFP (also see refs 25,26). Once the N-terminal region of Nrf1 is endoprotealytically cleaved, it would be represented as a processed 85-kDa protein. (**b**) COS-1 cells co-expressing Nrf1/GFP and the ER/DsRed marker were subjected to live-cell imaging combined with the *in vivo* membrane protease protection assay. The cells were permeabilized by digitonin (20 μg/ml) for 10 min, before being co-incubated with PK (50 μg/ml) for 30 min. During the course of the experiment, real-time images were acquired using the Leica DMI-6000 microscopy system. The merged images of Nrf1/GFP with ER/DsRed are placed (on *the third row of panels*), whereas changes in the intensity of their signals are shown graphically (*bottom*). The features of arrow-indicated cells are described in the main text. Overall, the images shown are a representative of at least three independent experiments undertaken on separate occasions that were each performed in triplicate (n = 9). (**c**) Additional live-cell imaging of Nrf1/GFP was acquired from 11 to 18 min after co-incubation of PK with digitonin, as described above.

As shown in Fig. 1a, it has been postulated that Nrf1 adopts various membrane-topologies^24^,^25^,^26^,^28^. To test this hypothesis, we performed further live-cell imaging of the Nrf1/GFP fusion protein combined with *in vivo* membrane protease protection assays, in order to determine whether the fusion protein is capable of being dislocated from within the ER lumen to the cyto/nucleoplasmic side of the ER membrane. In these experiments, COS-1 cells that had been co-transfected with expression constructs for Nrf1/GFP and the ER/DsRed marker were first pre-treated for 10 min with digitonin to pemeabilize cellular membranes before being challenged with proteinase K (PK) for 5–30 min (in the presence of digitonin) to digest cytoplasmic proteins; we envisaged that if Nrf1/GFP was transferred from the ER lumen to the cytoplasmic side of the membrane, it would become vulnerable to digestion by PK. As anticipated, the green fluorescent signal from Nrf1/GFP appeared to be superimposed upon red fluorescent images presented by ER/DsRed (Fig. 1b). No apparent change in the intensity of the green signal was observed within 10 min after treatment of the cells first with digitonin before being treated with PK for 10 min. By contrast, the green signal arising from the DsRed-GFP control protein was greatly diminished after 3 min incubation with PK and was completely degraded by 10 min of PK treatment (Fig. S2). Interestingly, several ‘hernia-like’ vesicles were observed to protrude from the cytoplasm of Nrf1/GFP-expressing cells that had been treated with PK for 11 min (indicated by *arrow* in Fig. 1c). Subsequently, these ‘hernia-like’ vesicles, along with the green images for Nrf1/GFP, gradually disappeared between 12 min and 17 min of treatment with PK (Fig. 1c). As expected, the image for ER/DsRed did not change within 18 min of PK treatment. When the cells were incubated with PK for 30 min, the ER/DsRed signal became weaker but did not disappear until they were exposed to Triton X-100 (i.e. a non-ionic surfactant used to solubilize membrane lipids). Collectively, these observations suggest that Nrf1/GFP is located primarily in the ER. They are also consistent with the notion that the ER-resident fraction of Nrf1/GFP can become dynamically repartitioned out of the organelle and retrotranslocated from the luminal side of the membranes into the cyto/nucleoplasmic side, whereupon it would become vulnerable to digestion by PK. We also noted that a small fraction of the cytosolic Nrf1/GFP that was not completely digested by PK could diffuse across permeabilized plasma membranes into the extracellular environment.

In a parallel control imaging experiment of DsRed-GFP, the double fluorescent intensity of DsRed and GFP, which was principally located in the cytoplasm of cells (on the *left-handed* side, Fig. S2) was decreased by about 25% and 50% between 5 min and 10 min after incubation with digitonin, respectively (Fig. S2, *the second lower graphs*). The remaining signals from DsRed and GFP were rapidly abolished by 1 min PK treatment and completely destroyed by 3 min PK treatment, suggesting that cytosolic DsRed-GFP is not protected by membranes against PK digestion. By contrast, the fluorescent signal from cells that relatively over-expressed DsRed-GFP (in *right upper cells*) was not diminished by digitonin. Indeed, their fluorescent signal seemed to be enhanced by 3 min of PK treatment because the cell was reduced by ~30% in size as compared to untreated cells (Fig. S2, *bottom graphs*). Thereafter, the DsRed-GFP signals were greatly diminished by PK treatment for 10 min, and completely disappeared by 15 min PK treatment, indicating that nuclear DsRed-GFP is incompletely protected by the nuclear envelope.

### Dynamic repartitioning of N275/GFP from the ER lumen into the cytoplasmic compartments

**Figure 2:**
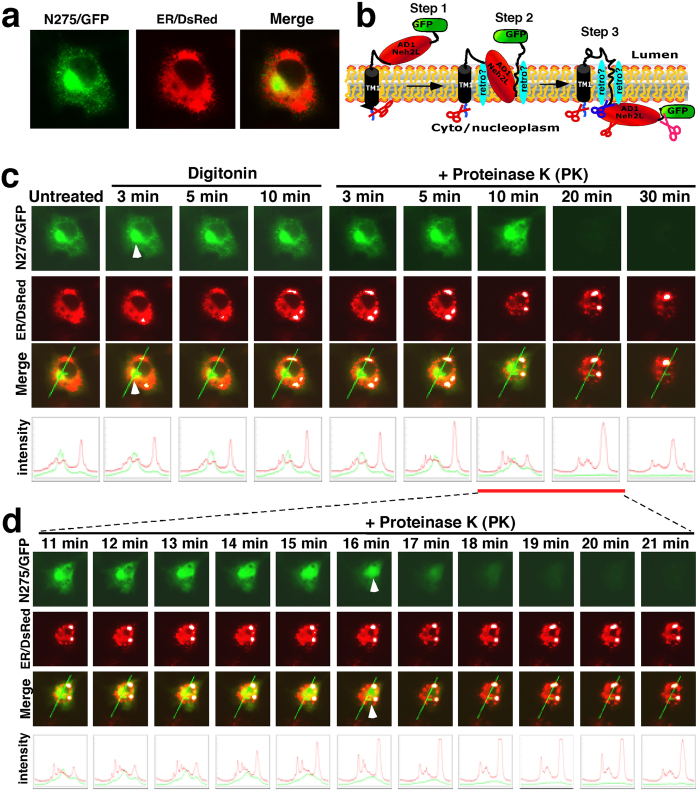
Live-cell imaging of N275/GFP that moves dynamically out of the ER membrane into the cytoplasm. (**a**) Localization of N275/GFP (in which the N275 portion contains the entire NTD and most of AD1 from Nrf1) within and around the ER. The merged images of Nrf1/GFP with ER/DsRed are also shown. (**b**) The putative dynamic membrane-topologies of N275/GFP are shown schematically. Within N275/GFP, the AD1 of Nrf1 has been fused C-terminally by GFP, which is postulated to face the ER luminal side of membranes during the initial topogenic vectorial process (see refs 25,26). (**c**) COS-1 cells co-expressing N275/GFP and the ER/DsRed marker were subjected to live-cell imaging combined with the *in vivo* membrane protease protection assay. During co-incubation of the cells with digitonon (20 μg/ml) and proteinase K (PK, 50 μg/ml) for 30 min, real-time live-cell images were acquired using the Leica DMI-6000 microscopy system. The merged images of Nrf1/GFP with ER/DsRed are placed (on *the third raw of panels*), whereas changes in the intensity of their signals are shown graphically (*bottom*). Overall, the images shown herein are a representative of at least three independent experiments undertaken on separate occasions that were each performed in triplicate (n = 9). (**d**) Additional live-cell imaging of Nrf1/GFP was acquired from 11 to 21 min after co-incubation of PK with digitonin, as described above.

Previous results obtained from mapping glycosylation sites within Nrf1 and from *in vitro* membrane protection assays suggested that AD1, along with the NST glycodomain, is co-translationally translocated into the ER^25^ (Fig. 2a). To further examine whether AD1 is dynamically partitioned out of the ER lumen and retrotranslocated across the membrane into cyto/nucloplasmic compartments (Fig. 2b), we performed real-time imaging of live cells co-expressing a construct containing the 275 N-terminal residues of Nrf1 fused with GFP (N275/GFP, in which the N275 portion includes the entire NTD and most of AD1), together with the ER/DsRed marker, followed by an *in vivo* membrane protease protection assay. In co-transfected COS-1 cells, N275/GFP and ER/DsRed presented obviously different images, in that when merged the membrane-bound N275/GFP signal did not completely superimpose upon the luminal-resident ER/DsRed signal (Fig. 2c, *the third row of images*). Addition of digitonin to the cell culture medium did not cause an obvious change in the imaging of either fluorescent fusion proteins when compared to those obtained from untreated cells (Fig. 2c, *bottom graphs*). Also, no further changes in the signal from either construct were observed within 5 min PK treatment (in the presence of digitonin). Interestingly, after 10 min treatment with PK, when the cells reduced in size, the intensity of the N275/GFP signal appeared more intense (Fig. 2c), suggesting that its C-terminal AD1 portion that is attached to GFP was initially positioned within the ER lumen in a manner that resulted in it being partially protected by the membrane against protease digestion.

When the PK digestion time was extended to 20 min, we observed that the signal from N275/GFP gradually became fainter (Fig. 2d, *the first row of images and bottom graphs*), and thereafter the residual green fluorescent protein completely disappeared. By contrast, the red fluorescent signal from ER/DsRed was retained unless the membrane was disrupted by treatment with Triton X-100 (Fig. 2d and unpublished data). Taken together with our previous work^25^, these observations are consistent with the hypothesis that membrane-bound N275/GFP (with its C-terminal portion facing the lumen) is capable of being transferred dynamically from the luminal side of ER membranes into cytoplasmic compartments, whereupon it becomes susceptible to protease attack. In addition, we also noted that a considerable fraction of N275/GFP was likely to be localized within juxtanuclear aggresome-like inclusion bodies within the ER (indicated by *arrow* in Fig. 2c), as described previously^32^, but the underlying mechanism needs to be elucidated.

### AD1 makes differential contributions to the stability of distinct Nrf1 isoforms

**Figure 3:**
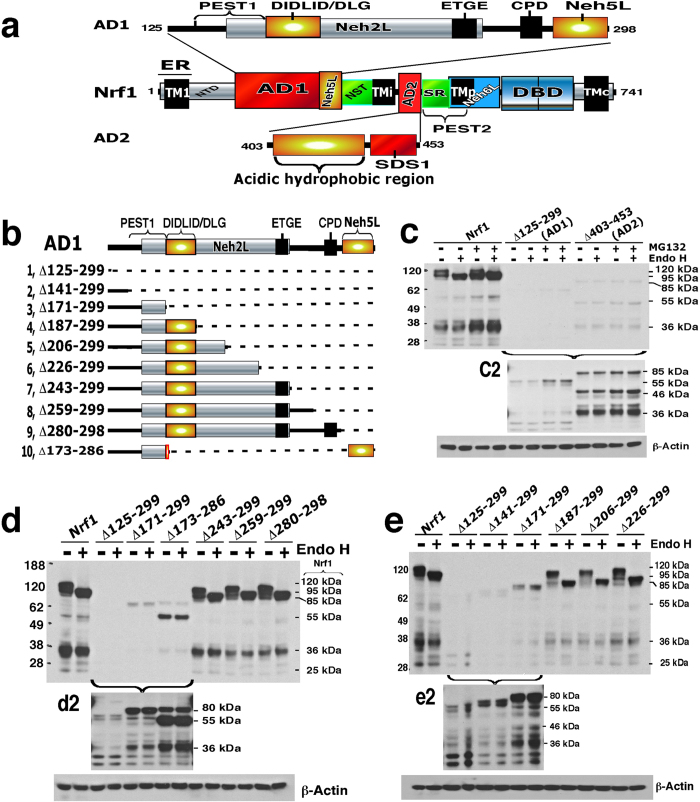
Nrf1 is differentially regulated by AD1, which is transiently translocated into the ER. **(a)** Diagrammatic representation of discrete regions in AD1 and AD2, as well as several putative degrons in Nrf1. Within the CNC-bZIP factor, two acidic transactivation domains AD1 (aa 125–298) and AD2 (aa 403–453) are separated by the NST glycodomain (aa 299–400) that contains the TMi peptide (aa 376–393) on its C-terminal border. This location suggests that the functioning of AD1 and AD2 could be monitored by TMi, with the topological folding and positioning of TMi being controlled by glycosylation and the stress-dependent non-glycosylation and/or de-glycosylation of its adjacent peptide sequences. Furthermore, the TMp region (aa 507–525) is situated on the C-terminal border of the SR/PEST2 sequence (aa 454–519) that overlaps the Neh6L domain (aa 489–580), indicating that the topology of TMp could exert an opposing effect on Nrf1 activity, thereby bidirectionally regulating target genes. (**b**) A series of deletion mutants lacking various portions of AD1 (shown by *hatched lines*) are illustrated schematically. The locations of the PEST1 sequence (aa 141–170), the DIDLID/DLG element (aa 171–186) and the ETGE motif within the Neh2L subdomain (aa 156–242), CPD (i.e. ^267^LLSPLLT^273^) and the Neh5L subdomain (aa 280–298) are indicated. (**c** to **e**) COS-1 cells were transfected with expression constructs for wild-type Nrf1 or the indicated mutants, and were then allowed to recover from transfection for 24  h in 25 mM-glucose medium, prior to being treated with 5 μmol/l MG132 for an additional 2 h. Total cell lysates (30 μg protein) were subjected to deglycosylation reactions with 500 units of Endo H at 37 °C for 1 h before being stopped in denaturation buffer. Thereafter, the abundance of these proteins was determined by immunoblotting with V5 antibody; the film was exposed to the immunoblot for two different lengths of time, with the longer exposed film being cropped to eliminate intense bands (*c2*, *d2* and *e2*). The masses of the Nrf1 isoforms was estimated to be 120, 95, 85, 55, 46, 36 and 25 kDa, and β-actin was employed as an internal control.

We recently found that the AD1 region in Nrf1 contributes to its basal transactivation activity as well as to its stimulation by glucose deprivation^8^,^25^. AD1 contains a potential PEST1-containing sequence (aa 125–170), the DIDLID/DLG element (aa 171–186), the Cdc4 phosphodegron (CPD, ^267^LLSPLLT^273^)-linker region (aa 261–279), the Neh2L subdomain (aa 156–242), and the Neh5L subdomain (aa 280–298) (see Fig. S1). To determine whether the potential degrons, PEST1, CPD and Neh2L, influence Nrf1 stability, we transfected COS-1 cells with a series of deletion constructs that lacked portions of the AD1 domain before treating them with the proteasome inhibitor MG132. Deletion of the entire AD1 (to yield Nrf1^Δ125–299^) yielded an unstable mutant glycoprotein, which was rapidly degraded to polypeptides of 60, 55, 36, 25 and 23 kDa (Fig. 3b–e), suggesting that AD1 is required for stability and correct folding of Nrf1 in the ER.

Western blotting showed that the N-terminal half of AD1, particularly the DIDLID/DLG-adjoining region, is required for the stability of Nrf1 protein (Fig. 3d,e). This is supported by evidence that either deletion of residues 173–286 (including most of Neh2L and CPD) (Fig. 3b, *construct 10*) or progressive loss of residues 125–186 (covering PEST1 and DIDLID/DLG) (*constructs 6 to 9*) resulted in the production of unstable non-glycosylated/de-glycosylated proteins of between 80-kDa and 55-kDa, that were further degraded into smaller polypeptides of between 55-kDa and 25-kDa (Fig. 3c2–e2; the 55-kDa protein is called LCR-F1/Nrf1β). Nonetheless, Nrf1 mutants lacking various portions between residues 187 and 299 (including the C-terminal two-thirds of Neh2L, the entire CPD and/or Neh5L regions) appeared more stable, and we did not observe a relative increase in the abundance of lower molecular mass Nrf1 isoforms (Fig. 3d,e).

**Figure 4:**
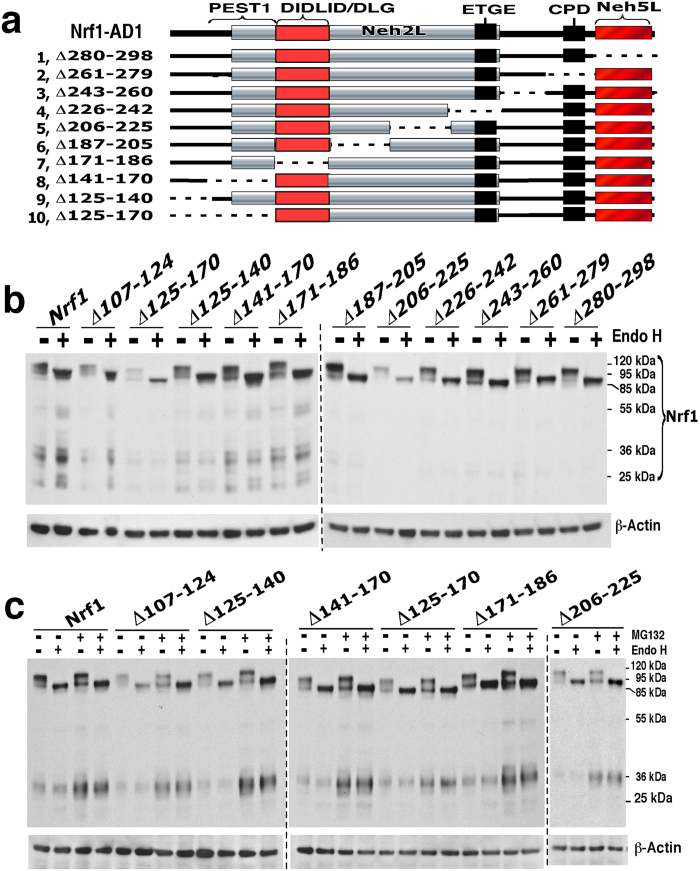
AD1 is required for Nrf1 stability and its glycosylation. (**a**) Discrete regions within AD1, including PEST1, Neh2L (containing the DIDLID/DLG element and ETGE motif), CPD (i.e. ^267^LLSPLLT^273^) and Neh5L, are shown (*upper cartoon*). A series of progressive deletions (shown by *lower hatched lines*) were created from within the AD1 (aa 125–298) of Nrf1. (**b**) COS-1 cells were transfected with an expression construct for Nrf1 or the indicated mutants. Total cell lysates were subjected to deglycosylation reactions with Endo H. The reactants were resolved by 4–12% LDS/NuPAGE and visualized by immunoblotting. Removal of PEST1 and its adjoining portions within Neh2L gave rise to unstable proteins when compared to wild-type Nrf1. (**c**) Following overnight recovery from transfection with expression constructs for Nrf1 or mutants, COS-1 cells were treated with 5 μmol/l MG132 for 2 h before being harvested. Subsequent deglycosylation products were examined by immunoblotting. The presence of aa 107–170 and aa 206–225 appears to increase the basal abundance of Nrf1, and MG132 treatment did not obviously increase the levels of the 120-kDa mutant glycoprotein rather than the 95-kDa protein, indicating that the 120-kDa mutant proteins are subject to proteasome-independent proteolytic degradation.

Further examination of a series of internal AD1 deletions indicated that PEST1, Neh2L and the CPD-linker region do not act as degrons for the full-length 120-kDa Nrf1 glycoprotein (Fig. 4). While PEST1, Neh2L and CPD are transiently positioned within the ER lumen^25^,^26^, they do not appear to contribute to degradation of the full-length Nrf1 glycoprotein as the abundance of the 120-kDa protein band was not increased by their deletion (Fig. 4b,c). However, dislocation of AD1 from the ER lumen into the cyto/nucleoprotein enables PEST1 and CPD to become *bona fide* degrons such that both can target the 95-kDa non-glycosylated and/or deglycosylated Nrf1 protein to separate proteolytic degradation pathways^29^,^31^.

**Figure 5:**
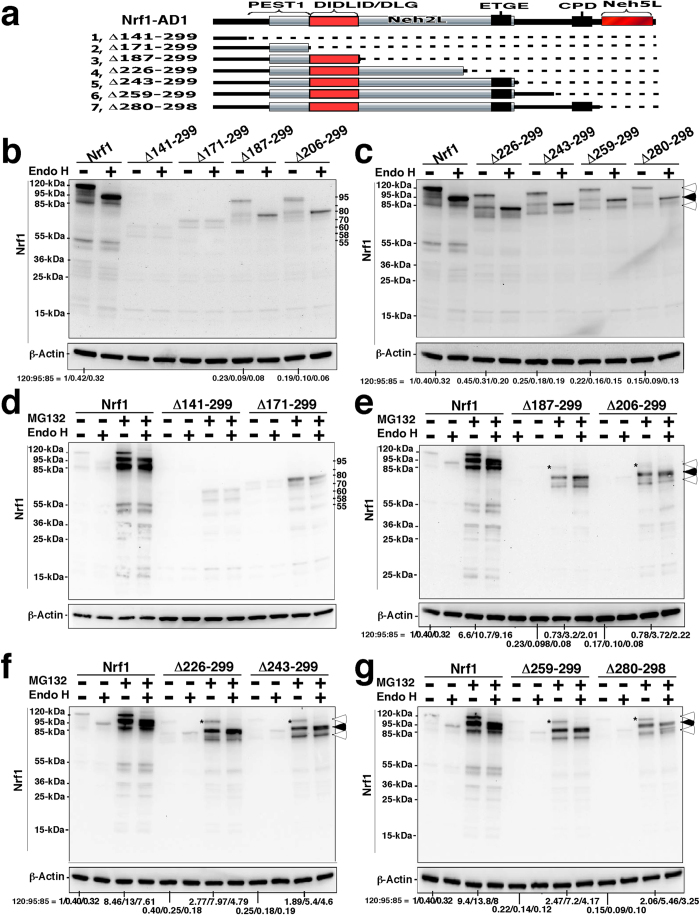
Stabilization of the 95-kDa and 120-kDa Nrf1 is differentially regulated by discrete regions within AD1. (**a**) Discrete regions within AD1, including PEST1, Neh2L (containing the DIDLID/DLG element and ETGE motif), CPD and Neh5L, are shown (*upper cartoon*). A series of progressive deletions (shown by *lower hatched lines*) were created from within the AD1 (aa 125–298) of Nrf1. (**b** to **g**) COS-1 cells expressing Nrf1 and mutants lacking various peptide sequences between aa 141–299 (covering most of PEST1, Neh2L, CPD and Neh5L) from within AD1, were treated with or without MG132 before being harvested. Total cell lysates were then subjected to deglycosylation reactions followed by western blotting. In the absence of Endo H, a major 120-kDa glycoprotein was detected along with the minor 95-kDa isoform and little of the 85-kDa protein (and other shorter isoforms indicated). However, MG132 treatment increased the abundance of the 120-, 95- and 85-kDa proteins, but with a more obvious increase in the latter two polypeptides. Notably, immunoblotting of both Nrf1^Δ141–299^ and Nrf1^Δ171–299^ showed that these two mutants gave rise to several non-glycosylated, deglycosylated and/or protealytically processed polypeptides of between 80 kDa and 36 kDa, and their abundance was increased by MG132. The intensity of the 120-, 95- and 85-kDa protein blots was estimated by dividing the value for all three Nrf1 isoforms with that for β-actin, and then the relative amount of all relevant Nrf1 isoforms (detected on the same gel) was normalized to the basal level (designated 1.0) of the 120-kDa protein measured from untreated cells, the results of which are shown in the *bottom*.

Following treatment with MG132, mutants lacking varying lengths of the C-terminal 112-aa portion of AD1 (covering the C-terminal half of Neh2L, CPD and Neh5L) increased the abundance of the ~95-kDa Nrf1 protein relative to that of the ~120-kDa glycoprotein (Fig. 5e,g), whilst the basal expression of ~120-kDa protein was relatively more than that of the ~95-kDa Nrf1 (Fig. 5b,c). Both basal and MG132-stimulated expression levels of the ~95-kDa and ~120-kDa isoforms were decreased by mutants lacking portions of CPD and Neh5L in AD1, when compared to their corresponding full-length proteins. These data suggest that other degrons besides PEST1 and CPD can also target the 95-kDa Nrf1 isoform for proteolysis in cyto/nucleoplasmic compartments *via* proteasome-dependent and/or -independent pathways. Furthermore, MG132 treatment of COS-1 cells expressing Nrf1^Δ125–299^ (lacking the entire AD1), Nrf1^Δ141–299^ (retaining the N-terminal half of PEST1 within AD1) or Nrf1^Δ171–299^ (retaining the entire PEST1 sequence) caused a marked increase in the abundance of non-glycosylated/deglycosylated polypeptides of 80-, 70-, 60-, 55- and 36-kDa (Figs 3c and 5d), indicating that other TAD-adjoining degrons [e.g. the DSGLS motif (aa 447-451) and the PEST2 sequence (aa 456-519)] can target Nrf1 protein for proteolytic degradation by the 26S proteasome.

### Differential regulation of Nrf1 activity by AD2, SR, Neh6L and their adjoining sequences

**Figure 6:**
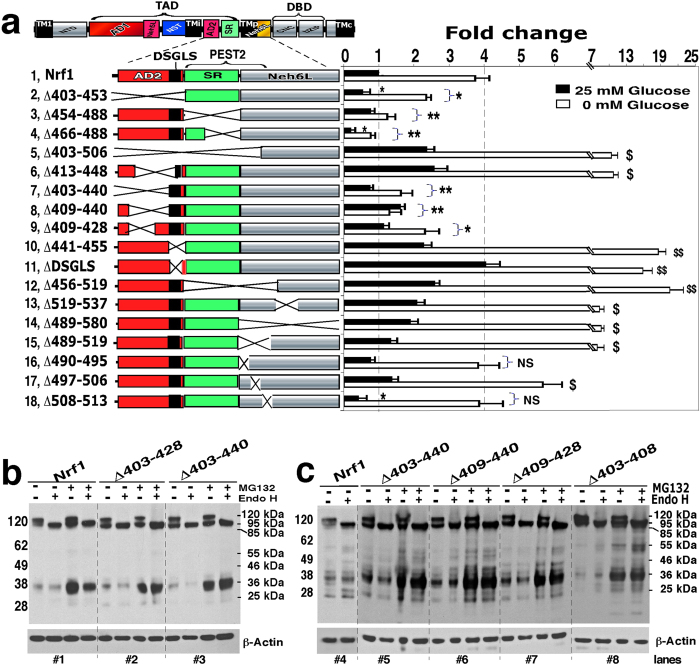
Differential regulation of Nrf1 by AD2, SR, PEST2, Neh6L and their adjacent degrons. (**a**) The *left* schematic shows Nrf1 deletion mutants lacking various portions within AD2, including DSGLS-SDS1, SR, PEST2-SDS2 and Neh6L (Figs. S1D and S3A). The contributions of these regions to increased Nrf1 activity in response to glucose starvation (i.e. glucose-free medium), when compared with Nrf1 activity in medium fortified with 25 mM-glucose (control), were examined using an ARE-driven reporter assay. COS-1 cells that had been cotransfected with one of the expression constructs (1.2 μg), together with *P*_*SV40*_*GSTA2-*6 × ARE-Luc (0.6 μg) and β-gal plasmid (0.2 μg), were allowed to recover from transfection in fresh 5.5 mM-glucose medium for 8 h, before being cultured in no-glucose medium for a further 18 h. Transactivation of ARE-driven luciferase gene by Nrf1 and its mutants was determined as described in the text. The data were calculated as a fold change (mean ± S.D) of transactivation mediated by wild-type Nrf1 under 25 mM-glucose control conditions. Significant increases (^$^p < 0.05 and ^$$^p < 0.001, n = 9) and decreases (*p < 0.05, **p < 0.001, n = 9) are indicated. (**b** and **c**) Cells expressing wild-type Nrf1, or mutants lacking the indicated portions of AD2, were allowed to recover from transfection for 24 h in 25-mM glucose-containing medium before treatment with 5 μmol/l MG132 for an additional 2 h. Following deglycosylation by Endo H, the proteins were resolved using LDS/NuPAGE containing 7% polyacromide gel in the pH 8.3 Tris-Acetate-SDS buffer and visualized by immunobloting with V5 antibodies. The amount of protein applied to each polyacrylamide-gel sample well was adjusted to ensure equal loading of β-gal activity. As indicated, the masses of the Nrf1 isoforms was estimated from their electrophoretic mobilities to be 120, 95, 85, 55, 46, 36 and 25 kDa, and β-actin was employed as an internal control.

In view of the fact that AD1 and the NST glycodomain did not appear to be fully responsible for the stimulation of Nrf1 activity caused by glucose deprivation^25^, we examined whether the proline-kinked hinge transmembrane region (TMp, aa 507–525)-adjoining regions in the Neh6L domain contribute to regulation of the transcription factor. Mutagenesis across AD2 (aa 403–453), SR (aa 454–488) and Neh6L (aa 489–580) regions produced four different effects (Fig. 6a). Firstly, the basal activity of Nrf1 and its stimulation by glucose deprivation were significantly decreased by deletion of AD2 (in Nrf1^Δ403–453^, *row 2*) and most of SR (in Nrf1^Δ466–488^*, row 4*), but not of Neh6L (in Nrf1^Δ489–580^*, row 14*). By contrast, loss of either various portions between aa 403–440 of AD2 (*rows 7, 8, 9*) or the entire SR region (in Nrf1^Δ454–488^*, row 3*) did not decrease basal Nrf1 activity, but diminished significantly its stimulation by glucose deprivation. Secondly, basal Nrf1 activity was partially repressed by deletion of the core TMp hydrophobic region (to yield Nrf1^Δ508–513^), whereas its stimulation by glucose deprivation was similar to that observed in wild-type Nrf1 (*cf. rows 18 with 1*). Thirdly, basal activity was not suppressed, but its stimulation by glucose deprivation was significantly increased, by loss of the TMp-adjoining SDS2 region (in Nrf1^Δ489–519^, *row 15* in Fig. 6a, and see Fig. S3) and the SDS2 core sequence (in Nrf1^Δ497–506^, *row 17*). Fourthly, the basal activity was increased and its stimulation by glucose deprivation was also substantially augmented by deletion of the entire AD2 plus the SR/PEST2 sequence (in Nrf1^Δ403–506^, Fig. 6a, *row 5*), as well as by deletion of either Neh6L (in Nrf1^Δ489–580^, *row 14*) or a TMp-adjacent region in Neh6L (in Nrf1^Δ519–537^, *row 13*).

Intriguingly, the increase in basal and glucose deprivation-stimulated Nrf1 activity was not associated with changes in glycosylation but was associated with absence of either the ^447^DSGLS^451^ motif (Nrf1^ΔDSGLS^, *row 11* in Fig. 6a) and its adjoining amino acids within AD2 (*rows 6* & *10*), or the adjacent PEST2 sequence (aa 456–519 covering the entire SR region and the SDS2 portion of Neh6L; *row 12*), suggesting that these regions restrain Nrf1 activity. Together, these data demonstrate that Nrf1 is subject to opposing regulation by sequences within AD2 and the SR/PEST2 regions: on the one hand, its full activity requires the presence of the intermediate transmembrane region (TMi, aa 367–393)-associated portions of AD2 and SR; but on the other hand, it is repressed by the DSGLS-containing SDS1 motif and the PEST2 degron (with their amino acid sequences shown in Fig. S1D and S3A). In addition to the TMp-adjoining SDS2 region, the potential ^554^DSAx_5_S^562^ degron, along with a potentially ubiquintinylated five-lysine cluster (K_5_-Ub, see Fig. S3A), may also contribute to negative regulation of Nrf1 by Neh6L, as its equivalent degron (DSAx_2_S) has been identified in Nrf2 (ref. 33).

### AD2-adjoining regions make distinct contributions to Nrf1 stability and transactivation activity

We next examined the molecular basis for control of Nrf1 stability through AD2 and its flanking SR and PEST2 sequences. Deletion of AD2 (giving Nrf1^Δ403–453^) resulted in the appearance of a putative unstable mutant protein that was rapidly degraded to generate several shorter polypeptides of between 85 kDa and 36 kDa (Fig. 3c and S4C). This indicates that AD2, like AD1, also contributes to the correct folding of Nrf1 and its proper topology within membranes.

Within AD2, deletion of the acidic-hydrophobic residues 403–440 (giving Nrf1^Δ403–440^) or residues 403–428 (giving Nrf1^Δ403–428^) caused a marked increase in the abundance of the 95-kDa protein rather than the 120-kDa glycoprotein or the 36-kDa Nrf1γ form (Fig. 6b, *cf. lanes* #*2,* #*3 with* #*1*). The increase in the 95-kDa protein was not further augmented by MG132 treatment, though the abundance of the 36-kDa form was significantly increased by the proteasome inhibitor. Surprisingly, the increase in the 95-kDa Nrf1^Δ403–440^ mutant protein was not accompanied by a corresponding increase in basal transactivation activity, when compared with the wild-type protein (Fig. 6a, *rows 7 vs 1*). By comparison with Nrf1^Δ403–440^, largely similar effects on protein abundance and transactivation activity were also observed with Nrf1^Δ409–440^, Nrf1^Δ409–428^ (lacking the core amphipathic AD2 portion), and Nrf1^Δ403–408^ (lacking a PxxPxP-hinged hexapeptide) (Fig. 6c, *cf. lanes* #*5 with* #*6 to* #*8*). These mutant proteins all lack various portions of the amphipathic residues 403–440 within AD2, but retain the putative DSGLS-containing SDS1 motif in this domain (Fig. S1D) and the flanking PEST2 sequence (Fig. S3A).

Notably, electrophoresis revealed that the Nrf1^Δ403–453^ mutant, which lacks the DSGLS motif but retains the PEST2 sequence, yielded a faint 85-kDa non-glycosylated/deglycosylated protein, and that neither the 95-kDa nor 120-kDa protein bands were observed (Fig. 3c and S4C). The 85-kDa Nrf1^Δ403–453^ protein band was accompanied by appearance of a 36-kDa polypeptide (called Nrf1γ) that lacks a TAD, and this is thought to be responsible for the significant decrease in transactivation activity (Fig. 6a, *row 2*). Treatment with MG132 did not increase the abundance of the 85-kDa Nrf1^Δ403–453^ protein. However, the abundance of smaller Nrf1 proteins (between 55-kDa and 36-kDa) was increased by MG132 treatment (Fig. 3c). Remarkably, deletion of AD2 plus the PEST2 degron (in Nrf1^Δ403–506^) allowed complete recovery of the 95-kDa and 120-kDa proteins, which was in sharp contrast to the results for Nrf1^Δ403–453^ (Fig. S4). Also, the Nrf1^Δ403–506^ mutant exhibited substantial transactivation activity (Fig. 6a, *row 5*). Similar results to those for Nrf1^Δ403–506^ were also observed for the Nrf1^Δ413–448^ mutant (which retains both the N-terminal ^403^PELPDPLGGL^412^ and C-terminal ^449^GLSLDSS^455^ peptides of AD2, see Fig. 6a, *row 6,* and S4B). Together, these data suggest that discrete portions of AD2, along with the flanking TMi region, the DSGLS-containing SDS1 motif and the PEST2 sequence, make distinct contributions to both the stability of Nrf1 protein and its transactivation activity.

### The putative AD2-adjoining SDS1 and PEST2 degrons monitor selective proteolytic processing of Nrf1 to yield the low-molecular-weight isoforms

Besides glycosylation and stability, the ability of Nrf1 to transactivate ARE-driven gene expression is also influenced by the formation of short isoforms of 55-kDa Nrf1β, 36-kDa Nrf1γ and/or 25-kDa Nrf1δ. Amongst them Nrf1β is a short-life protein with weak activity^8^,^34^,^35^,^36^, whilst Nrf1γ and Nrf1δ have been shown to have dominant-negative effects against the activity of intact wild-type Nrf1 and/or Nrf2 (refs 35,36); they are deduced to arise from the in-frame translation from an internal ATG codon within a longer Nrf1 transcript (Fig. S5) and may also be generated from proteolytic degradation of a larger Nrf1 isoform. To address the latter issue, herein we examine which AD2-adjoining degrons (i.e. DSGLS, SDS1, PEST2, and SDS2) monitor selective proteolytic processing of Nrf1 to yield the short isoforms.

**Figure 7:**
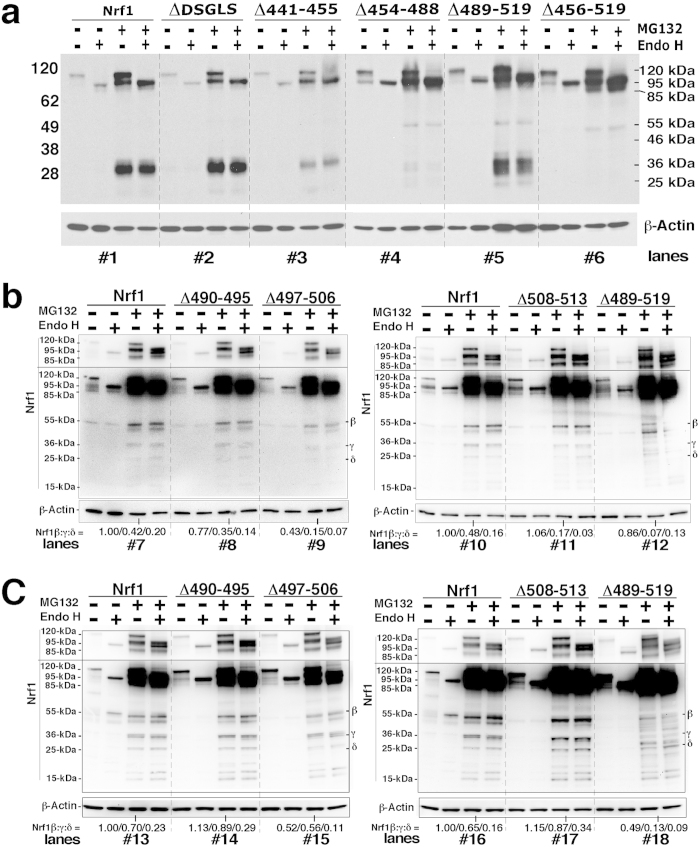
AD2-adjoining degrons selectively target Nrf1 for proteolytic processing into several small isoforms. (**a**) When COS-1 cells were grown to reach ~90% of confluence, they were transfected overnight (for 18 h) with expression constructs for the intact wild-type Nrf1 or the indicated mutants lacking DSGLS, SDS1, SDS2, SR and PEST2, and then were allowed to recover from transfection for an additional 24 h in 25-mM glucose-containing medium before being treated with 5 μmol/l MG132 for 2 h prior to the termination of experiment. After *in vitro* deglycosylation reactions of denatured lysates with Endo H, the proteins were resolved using LDS/NuPAGE containing 4–12% Bis(polyacrylamide)-Tris gel in the pH 7.3 MES-SDS running buffer, and the blots probed with antibodies against the V5 epitope. The amount of proteins applied to each polyacrylamide gel sample well was adjusted to ensure equal loading of β-gal activity. In addition, β-actin was employed as an internal control of protein loading. (**b** and **c**) When COS-1 cells were grown to reach either ~50% (**b**) or ~70% (**c**) of confluences, they were transfected with expression constructs for the intact wild-type Nrf1 or the indicated mutants lacking SDS2 and its flanking peptides for 6 h (**b**) or 12 h (**c**). The cells were then allowed to recover from transfection for an additional 24 h in 25-mM glucose-containing medium before being treated with 5 μmol/l MG132 for 2 h. The total lysates were subjected to deglycosylation reactions, followed by protein separation by LDS/NuPAGE that contains 4–12% Bis-Tris gel in the pH 7.7 MOPS-SDS running buffers before being visualized by western blotting. The *upper* and *middle* images were presented for different times of exposure to the supersensitive reagent. Subsequently, the intensity of the 55-, 36- and 25-kDa protein blots was estimated by dividing the value for Nrf1 isoforms with that for β-actin, and then the relative amount of all relevant Nrf1 isoforms (detected on the same gel) was normalized to the basal level (designated 1.0) of the 55-kDa protein measured from MG132-treated cells expressing wild-type Nrf1, the results of which are shown in the *bottom*.

In COS-1 cells that had been transfected with Nrf1^Δ441–455^, lack of the SDS1 sequence that includes the DSGLS motif caused an increase in the basal expression of the 36-kDa Nrf1γ protein, rather than 25-kDa Nrf1δ (Fig. S4,C & E). This was accompanied by modest amounts of the 85-kDa, 95-kDa and 120-kDa proteins resulting in a ~2.2-fold increase in basal transactivation activity (Fig. 6a, *rows 10 vs 1*), that was not blocked by high expression of 36-kDa Nrf1γ. This observation suggests that SDS1 negatively regulates wild-type Nrf1 possibly through the DSGLS degron targeting the protein to proteasome-mediated degradation *via* β-TrCP, as consistent with previous report^29^. Unexpectedly, accumulation of Nrf1γ by the proteasomal inhibitor MG132 was not as pronounced for the Nrf1^Δ441–455^ mutant as it was for the Nrf1^ΔDSGLS^ mutant (which retained its EEEFDSLDSS residues) (Fig. 7a, *cf. lanes* #*2,* #*3 with* #*1*), but neither mutant caused an increase in the abundance of the 95-kDa and 120-kDa Nrf1 proteins. This finding indicates that besides the DSGLS degron, the acidic decapeptide (EEEFDSLDSS) within SDS1 also enables Nrf1 to be targeted for proteasome-dependent proteolytic processing to generate the Nrf1γ protein. However, the difference between Nrf1^Δ441–455^ and Nrf1^ΔDSGLS^ in accumulation of Nrf1γ stimulated by MG132 did not affect the ability of glucose deprivation to induce ARE-driven luciferase activity ~15-fold (Fig. 6a, *rows 10 vs 11*); under basal conditions, Nrf1^ΔDSGLS^ supported a ~4-fold increase in reporter gene activity, whilst Nrf1^Δ441–455^ only supported ~2.2-fold activity (Fig. 6a, *cf. rows 10 with 11*). Collectively, these seemingly paradoxical results suggest that the ~36-kDa Nrf1γ does not appear to compete against the activity of Nrf1 mutants lacking the DSGLS degron; in turn, it cannot be ruled out that the DSGLS-containing SDS1 degron is likely to target the protein to a proteasome-independent proteolytic pathway, in order to yield a ~36-kDa protein, the stability of which would rather be regulated by the proteasome-dependent proteolytic degradation pathway. Together with the topology of Nrf1, we envisage that differential or opposable regulation of the intact Nrf1 factor by the DSGLS-containing SDS1 motif at dually controlling protein stability and transactivation activity may also depend on dynamic topovectorial positioning of the degrons that flank AD2 and the PEST2 sequence (both contribute to positive and negative regulation of Nrf1, respectively) around and within membranes.

Deletion of the entire PEST2 sequence (in Nrf1^Δ456–519^, Fig. S4E), its N-terminal SR subdomain (in Nrf1^Δ454–488^, Fig. S4B and C) or its C-terminal SDS2 region (in Nrf1^Δ489–519^, Fig. S3B, C) caused a marginal reduction in the basal levels of the 36-kDa Nrf1γ protein, leading to a modest increase in the longer 95-kDa and 120-kDa Nrf1 isoforms. Importantly, accumulation of Nrf1γ by MG132 was almost completely prevented by loss of the entire PEST2 sequence (Fig. 7a, *lanes* #*6*) or the SR subdomain (Fig. 7a, *lanes* #*4*), and was also suppressed partially by loss of the SDS2 region (Fig. 7b, *lanes* #*12* and 7c, *lanes* #*18*, which was resolved on 4–12% NuPAGE Bis-Tris gels in the pH 7.7 MOPS-SDS running buffer, with an exception of no obvious change in the presence of Nrf1γ in Fig. 7a, *lanes* #*5*, which was separated on 4–12% NuPAGE Bis-Tris gels in the pH 7.3 MES-SDS running buffer). By contrast, the relative abundance of the 95-kDa and 120-kDa isoforms was increased in the case of all three Nrf1 mutants upon MG132 treatment (Fig. 7a). Failure to produce the low molecular weight protein seemed to be associated with a significant increase in the transactivation activity of the Nrf1^Δ456–519^ mutant lacking the PEST2 degron, but not Nrf1^Δ489–519^ (it still exhibited a modestly elevated activity although expression of Nrf1γ was partially retained) or Nrf1^Δ454–488^ (its basal activity appeared to be unaltered by the failure to yield Nrf1γ, but its glucose deprivation-inducible activity was significantly suppressed) (Fig. 6a, *cf. rows 12, 17 with 3*). These seemingly paradoxical results from Nrf1^Δ489–519^ and Nrf1^Δ454–488^ demonstrate that the presence of Nrf1γ is not allowed to inhibit competitively against the modest activity of the Nrf1^Δ489–519^ mutant lacking putative SDS2 degron, whereas the absence of Nrf1γ is not enabled to stimulate the activity of the Nrf1^Δ454–488^ mutant lacking the SR subdomain. Conversely, the fact that Nrf1^Δ454–488^ exhibited a significant low activity, though its failure to generate the ~36-kDa Nrf1γ at lower levels was accompanied by higher levels of the 95-kDa and 120-kDa isoforms, demonstrates that the SR portion is preferred to positive regulation of wild-type Nrf1, rather than negative contribution to the CNC-bZIP factor by the PEST2 degron. Together with the local topology of Nrf1, we surmise that differential or bidirectional regulation of Nrf1 by the entire PEST2 sequence, the SR and SDS2 portions may occur possibly *via* different topological mechanisms that control protein stability and its transactivation activity.

Overall, our findings unravel that within the PEST2 sequence, the SR subdomain may dually regulate Nrf1 because this subdomain contributes to inducible transactivation and serves to direct Nrf1 for proteasome-mediated proteolytic processing, thereby yielding the low-molecular-weight polypeptides between 55-kDa and 25-kDa. Besides the SR subdomain, the adjacent SDS2 region within PEST2 negatively regulates Nrf1 possibly through both proteasome-dependent and -independent proteolytic degradation pathways; this is due to variations in expression levels of the small isoforms arising from Nrf1^Δ489–519^ under different experimental conditions (Fig. 7, *cf*. *lanes* #*5 with* #*12 and* #*18*, and see Fig. S3, *cf*. *lanes* #*5 with* #*10*).

### Distinct contributions of the SDS2 region to the PEST2-directed proteolytic processing of Nrf1 through proteasome-dependent and/or -independent degradation pathways

Intriguingly, western blotting revealed variations in the expression pattern of short Nrf1 isoforms between 55-kDa and 25-kDa arising from the Nrf1^Δ489–519^ mutant (lacking the entire SDS2 region) and the intact prototypical form, is attributable to distinct experimental settings of COS-1 cells, with different passages (e.g. from 22 to 35) and confluences (e.g. between 50% and 90%), that had been transfected for 6 h (Fig. 7b and S3B), 12 h (Fig. 7c and S3C), or 18 h (Fig. 7a) before being recovered for additional 18 h (Figs 6c & 7a) or 24 h (Fig. 7b,c). In addition, certain alterations in the amount of short Nrf1 isoforms and their electrophoretic mobility were also influenced by different experimental procedures of LDS/NuPAGE that contains: i) 4-12% Bis-Tris gel in different running buffers [i.e. the pH 7.3 MES-SDS (Fig. 7a) or the pH 7.7 MOPS-SDS (Fig. 7b,c and S3B,C)]; ii) 10% polyacrylamide Bis-Tris gel in the pH 8.9 Tris-glycine-SDS running buffer (Fig. S4D); or iii) 7% polyacromide Tris-Acetate (TA) gel in the pH 8.3 TA-SDS buffer (Fig. 6c), beyond distinctive sensitivity of development substrates to immunoblots of Nrf1 polypeptides for distinct exposure times indicated.

Herein, total lysates of COS-1 cells that had been transfected for either 6 h (Fig. 7b and S3B) or 12 h (Fig. 7c and S3C) before being recovered for additional 24 h in a fresh complete medium, were subjected to a relatively clear protein separation by NuPAGE containing 4–12% Bis-Tris gel in the pH 7.7 MOPS-SDS running buffer, to provide a better understanding of the PEST2 degron targeting Nrf1 to distinct proteolytic degradation pathways. Close comparison of internal deletion mutants from within the PEST2 sequence with the wild-type factor revealed that the basal expression of the 55-kDa Nrf1β, 36-kDa Nrf1γ and/or 25-kDa Nrf1δ (Fig. S3, *lanes* #*5 & 10*) and their accumulation stimulated by MG132 (Fig. 7, *lanes* #*12 & 18*) were obviously prevented by the Nrf1^Δ489–519^ mutant, but it instead increased 120-, 95-, 85-, and 46-kDa isoforms to varying extents, such that loss of the entire SDS2 region enabled the mutant factor to exhibit a considerably elevated activity under the basal and glucose deprivation conditions (Fig. 6a, *row 15*). Further examination unravelled that loss of the SDS2 core motif (to yield the Nrf1^Δ497–506^ mutant) rendered it to partially prevent production of Nrf1γ and/or Nrf1δ under basal conditions (Fig. S3, *lanes* #*3 & 8*) and MG132-stimulated conditions (Fig. 7, *lanes* #*9 & 15*). The MG132-stimulated, rather than basal, expression of 55-kDa Nrf1β was also partially suppressed by the Nrf1^Δ497–506^ mutant (Fig. 7 & S3). Consequently, the partial prevention of these short Nrf1 polypeptides appears to be associated with a modest increase in abundance of the larger 85-kDa, 95-kDa and 120-kDa isoforms under the basal rather than MG132-stimulated conditions (Fig. S3). Accumulation of these longer isoforms enabled the Nrf1^Δ497–506^ mutant to increase both basal and inducible transactivation of ARE-driven reporter gene expression when compared with that observed with wild-type Nrf1 (Fig. 6a, *rows 17 vs 1*). These findings indicate that SDS2 may direct the selective proteolytic processing of Nrf1 to generate its small isoforms probably through proteasome-dependent and/or -independent pathways (such as that provided by calpain).

By comparison with wild-type Nrf1, the Nrf1^Δ490–495^ and Nrf1^Δ508–513^ mutants differentially caused marginal decreases in basal abundance of the shorter 55-kDa, 36-kDa and/or 25-kDa isoforms, as was accompanied by modest increases in the basal expression of the longer 120-kDa, 95-kDa and 85-kDa proteins, particularly during the transfection setting for a longer time (Fig. S3C). However, MG132-stimulated accumulation of almost all Nrf1 isoforms between 120-kDa and 25-kDa was roughly unaffected by Nrf1^Δ490–495^ or Nrf1^Δ508–513^ mutants (Fig. 7b,c), which also have nor obvious effects on their transactivation activity (Fig. 6a, *rows 16 vs 18*), with the exception that a marginal decrease in basal reporter gene activity mediated by Nrf1^Δ508–513^ was observed when compared with that mediated by full-length Nrf1 (Fig. 6a, *rows 18 vs 1*). Together with the above data from Nrf1^Δ456–519^ (lacking the PEST2 sequence), Nrf1^Δ489–519^ (lacking the SDS2 region with the TMp being located at its C-terminus of PEST2) and Nrf1^Δ497–506^ (lacking the core SDS2 region), these results led us to an assumption that different parts of the TMp-adjoining SDS2 region (i.e. aa 490–495, 497–506 and 508–513) might play dual opposing roles in stabilizing Nrf1; one region might restrict, though it could not be ruled out that another could facilitate, the putative degron activity of the PEST2 sequence targeting the intact wild-type full-length protein to proteasome-dependent or -independent proteolytic processing to produce the small isoforms. The selective processing of Nrf1 might also be monitored by its dynamic membrane-topological processes that account for the vectorial positioning and repartitioning of the putative degrons within and around the ER.

In addition, accumulation of the 36-kDa Nrf1γ and/or 25-kDa Nrf1δ proteins was also, to a greater or lesser extent, prevented by other deletion mutations such as Nrf1^Δ403–506^ (lacking both the entire AD2 region and the PEST2 sequence), Nrf1^Δ489–580^ (lacking the entire Neh6L domain) or Nrf1^Δ519–537^ (lacking a TMp-flanking peptide in the Neh6L domain). In these instances, the failure of low molecular weight proteins to accumulate was accompanied by an increase in abundance of the 95-kDa and 120-kDa isoforms (Fig. S4B–D); this was also associated with significantly augmented transactivation of an ARE-driven reporter gene (Fig. 6a, *rows 5, 13, 14*). These data indicate that Neh6L contributes to negative regulation of Nrf1 through other degrons (e.g. DSAx_5_S), besides the PEST2 sequence.

### Proteasome inhibitors exert both positive and negative effects on Nrf1 activity

**Figure 8:**
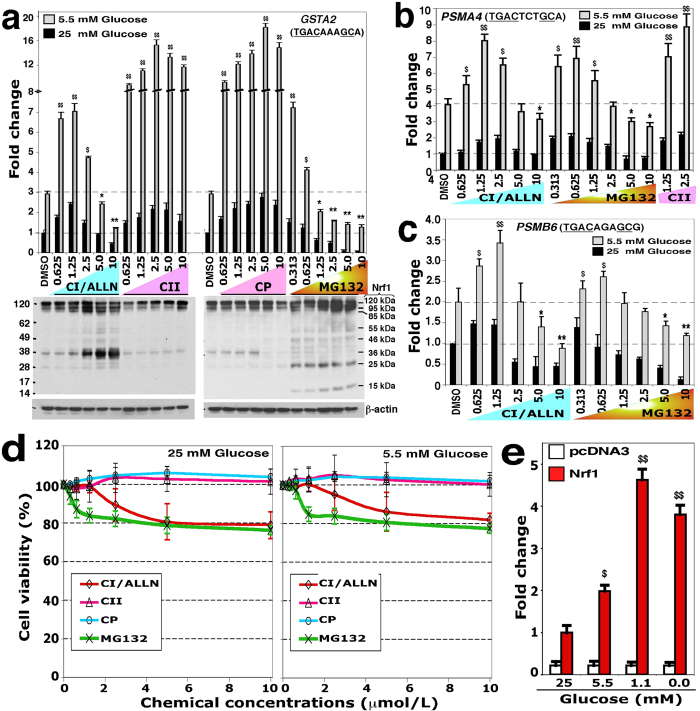
Bidirectional regulation of Nrf1 by proteasome- and/or calpain inhibitors to control target gene expression. (**a**) After 6-h transfection with Nrf1, *GSTA2-*6 × ARE-Luc and β-gal constructs, COS-1 cells were allowed to recover overnight in 5.5 mM-glucose medium before being treated for 18 h with MG132, CI/ALLN), CII, or CP at the indicated doses (μmol/l for MG132; μg/ml for the other three chemicals) in 5.5 or 25 mM-glucose medium. Thereafter, Nrf1-mediated activity (*upper*) was measured and is shown as a fold change against the vehicle value (designated 1.0). By comparison with the activity obtained from cells grown in medium containing 25 mM-glucose, significant increases (^$^p < 0.05; ^$$^p < 0.001, n = 9) or significant decreases (*p < 0.05 and **p < 0.001, n = 9) in reporter activity are indicated. The abundance of Nrf1 isoforms was examined by immunoblotting, and the results are shown from cells cultured in 25 mM-glucose (*middle*) or 5.5 mM-glucose media (Fig. S6B). (**b**) Bidirectional regulation of Nrf1 by proteasome and calpain inhibitors as assessed by measuring *PMSA4*/ARE-driven reporter activity. COS-1 cells expressing Nrf1, 3 × *PMSA4*-ARE-Luc (3 × *PMSA4*-mutARE-Luc as a negative control)^3^,^30^ and β-gal, were treated with the indicated doses of chemicals, followed by statistical analysis of luciferase activity, as described above. (**c**) Dual regulation of a CAT reporter gene by CI/ALLN and MG132. COS-1 cells expressing Nrf1, the reporter pCAT1500 (which contains 1500 bp of the promoter of *PMSB6* ligated to the *CAT* gene^2^; the ARE-Mut17 served as a negative control) and β-gal, were treated with the indicated doses of chemicals as described above. CAT activity was measured and compared against that for β-gal activity. (**d**) The cytotoxic effect of chemicals on COS-1 cells was assayed as described in the Materials and Methods section. (**e**) The activity of Nrf1 in response to glucose starvation was determined using the *GSTA2-*6 × ARE-Luc reporter assay, after an 8-h recovery of the transfected cells in 5.5 mM glucose-containing medium, and an additional 18-h during which time they were grown in medium containing the indicated glucose concentrations. Ectopic Nrf1-forced reporter gene activity was calculated as a ratio of its value against the background activity mediated by endogenous Nrf1 and/or Nrf2 (i.e. pcDNA3).

As shown in Fig. 8a (*upper*), we found that MG132 exerts distinct dose-dependent effects on Nrf1. At low doses (0.313 μmol/l), MG132 did not obviously influence basal Nrf1 activity (i.e. only ~1.5-fold measured in cells grown in medium containing 25 mM glucose), but increased Nrf1 activity ~7-fold when cells were grown in 5.5 mM glucose medium (Fig. 8a, *upper right panel*). This increase in ARE-driven reporter activity was accompanied by a modest increase in abundance of the 95-kDa Nrf1 protein, along with a decrease in abundance of Nrf1 polypeptides of between 15 kDa and 36 kDa (Fig. 8a and S6B, *right panels*). Relative to treatment with 0.313 μmol/l MG132, treatment of cells with 0.625 μmol/l MG132 allowed modest accumulation of the smaller Nrf1 isoforms such that its transactivation activity was decreased to ~4-fold (as compared with~7-fold) that could be mediated by its longer 95-kDa isoform. At higher doses of MG132 (1.25 to 10 μmol/l, with no obvious cytotoxity, see Fig. 8d), both basal Nrf1 activity and that stimulated by 5.5 mM glucose were gradually decreased, as the dose of MG132 was increased from 1.25 μmol/l to 10 μmol/l, by concomitantly increasing abundance of the smaller dominant-negative forms, although the amounts of the 120-, 95- and 85-kDa proteins also increased (Fig. 8a and S6B, *right panels*). A similar biphasic dose-dependent effect on Nrf1 was also found for calpain inhibitor-I (CI/ALLN, which also acts as a proteasome inhibitor^8^,^31^), with the decrease in ARE-driven reporter gene activity being associated with an increase in the level of the dominant-negative 36-kDa Nrf1γ (Fig. 8a, and S6B, *left panels*).

Treatment of cells with low doses of MG132 (0.313 and 0.625 μmol/l) or CI/ALLN (0.625 and 1.25 μg/ml) stimulated an increase in the activity of ectopic Nrf1 under normal and low glucose conditions, as measured by luciferase reporter constructs designed around the proteosomal *PSMA4* and *PSMB6* subunit genes (Fig. 8b,c). Conversely, activation of these two reporter genes by ectopic Nrf1 was gradually repressed by increasingly high doses of either MG132 (from 5 μmol/l to 10 μmol/l) or of CI/ALLN (from 5 μg/ml to 10 μg/ml). These results, taken together with those of other groups showing that Nrf1 and its longer TCF11 form increase the transcriptional expression of almost all proteosomal subunits (including PSMA4 and PSMB6) in the ‘bounce-back’ response to its inhibition by a low dose of MG132 or other proteosomal inhibitors^2^,^3^, indicate that a bidirectional regulatory feedback circuit exists between Nrf1 and its target proteasomal genes.

Cell viability assays showed that neither MG132 (at 0.313 to 1.25 μmol/l) nor CI/ALLN (at 0.625 to 5.0 μg/ml) were cytotoxic to COS-1 cells cultured in media containing 5.5 mM glucose or 25 mM glucose (Fig. 8d), and even higher doses of MG132 (5 μmol/l to 10 μmol/l) and CI/ALLN (10 μg/ml) were not obviously cytotoxic. These results indicate that the cytotoxicity of both proteosomal inhibitors does not predominantly contribute to their dose-dependent positive and negative regulatory effects on Nrf1-target reporter gene activity. Such activity was stimulated to varying degrees by glucose starvation (Fig. 8e). An approximate 3.8- or 4.6-fold increase in Nrf1-mediated activity was induced in COS-1 cells that were cultured in media containing zero or 1.1 mM glucose (Fig. 8e); this was accompanied by electrophoretic migration of Nrf1 as a major 95-kDa protein that represents either the non-glycosylated or the deglycosylated CNC/bZIP factor^25^.

### Activation of the Nrf1-mediated transcriptional response by calpain inhibitors

The dual calpain and proteasome inhibitor CI/ALLN, like MG132, has a biphasic dose-dependent effect on Nrf1 as described above (Fig. 8a, and S6B, *left panels*). Next, we further examined whether the calpain inhibitor-II (CII) and calpeptin (CP) have effects on Nrf1 that are similar to those exerted by CI/ALLN. As shown in Fig. 8a (*upper left panel*), the Nrf1 activity stimulated by growth in medium containing 2.5 mM glucose was gradually further increased from ~9-fold to ~18-fold as the concentration of CII was increased from 0.625 μg/ml to 2.5 μg/ml. However, additional increases in CII concentration from 2.5 μg/ml to 10 μg/ml brought about a modest reduction in Nrf1 activity to ~12-fold. Similarly, progressive increases in the concentration of CP from 0.625 μg/ml to 2.5 μg/ml also caused a gradual increase in Nrf1-mediated transactivation of ARE-driven luciferase reporter activity from ~9-fold to ~19-fold. The latter maximum Nrf1 activity was stimulated by 5.0 μg/ml CP and then decreased to ~17-fold activation by 10.0 μg/ml CP (Fig. 8a, *upper right panel*).

Western blotting showed that CII and CP caused modest increases in the 120-kDa Nrf1 glycoprotein, with an exception of 10 μg/ml CP, which caused a decrease in expression of both 120 kDa and 95 kDa isoforms in COS-1 cells grown in medium containing 25 mM glucose (Fig. 8a, *lower right panel*). By contrast, no apparent change in the 120-kDa and 95-kDa Nrf1 proteins was observed in CP-treated cells that had been grown in medium containing 5.5 mM glucose (Fig. S6B, *right panels*). However, treatment with CII and CP in cells that had been grown in medium containing either 25-mM or 5.5-mM glucose resulted in lower amounts of the 36-kDa Nrf1γ (Fig. 8a, *lower right,* and S6B). Moreover, CP did not further stimulate additional transactivation of a luciferase reporter gene mediated by mutant Nrf1 proteins that lacked the DSGLS-adjoing SDS1 motif, the PEST2 sequence, the SR and/or SDS2 regions (Fig. S6A), and they would as a consequence diminish the level of Nrf1γ (Fig. 7, S3 & S4). Interestingly, the cleaved 85-kDa Nrf1 protein was not observed in the CII- and CP-treated cells, and less 36-kDa Nrf1γ was apparent (Figs. 8a & S6B), when compared to MG132- and CI/ALLN-treated cells.

Further examination revealed that two doses of CII (1.25 and 2.5 μg/ml) also caused a significant increase in transcriptional activity of 3 × *PSMA4*-ARE-Luc mediated by Nrf1 (Fig. 8b), suggesting that calpain inhibitor also causes a similar response to that of the proteasomal inhibitors MG132 and CI/ALL, leading to transcriptional expression of proteosomal genes. Together with the above data from calpain and proteasomal inhibitors, we surmise that Nrf1-mediated transcriptional response to calpain inhibitors might be similar to an effect of proteasomal inhibitors on Nrf1, and this response could be induced through an uncharacterized pathway which enables selective proteolytic processing of Nrf1 to yield active or dominant-negative isoforms of between 85 kDa and 15 kDa. It should also be noted that the cleaved 85-kDa isoform of Nrf1 may also be produced through endoproteolytic processing of the CNC-bZIP protein by other proteases to remove its portions from its N-terminal region.

### Production of a cleaved 85-kDa Nrf1 protein is blocked by deletion of the ER luminal-anchoring NHB2 sequence

**Figure 9:**
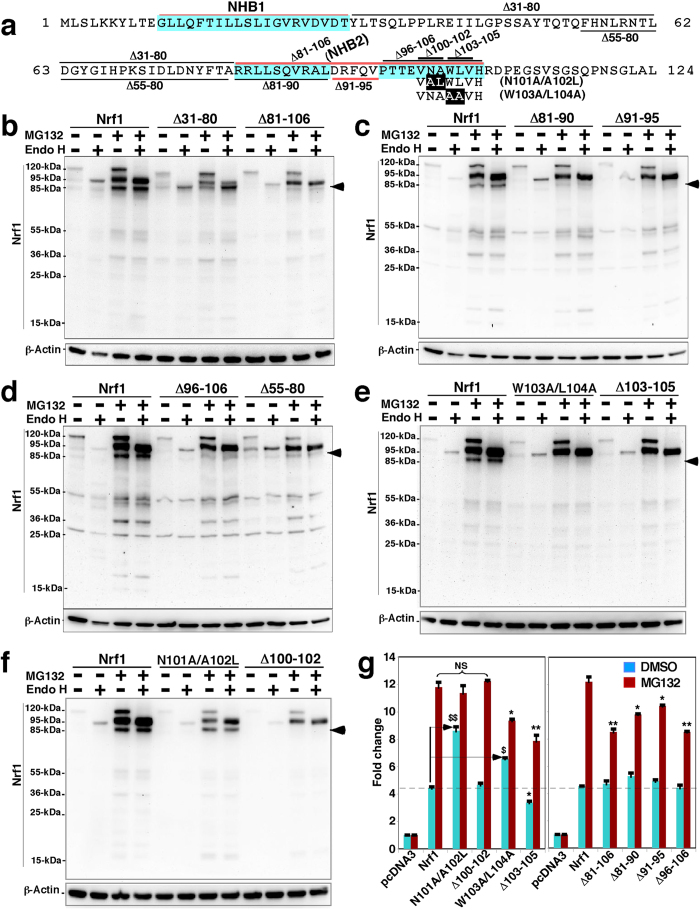
The abundance of cleaved 85-kDa Nrf1 protein is diminished by mutation of discrete regions around NHB2. (**a**) Schematic representation of discrete regions, including NHB1 (aa 11–30) and NHB2 (aa 81–106), as well as several mutants (made herein), within the N-terminal domain (NTD, aa 1–124) of Nrf1. (**b** to **f**) Each expression construct for wild-type Nrf1, or the indicated NTD deletion mutants, was transfected into COS-1 cells for 6 h. Thereafter, the cells were allowed to recover from transfection for 24 h in 25-mM glucose-containing medium before being treated with 5 μmol/l MG132 for an additional 4 h prior to termination of the experiment. After *in vitro* deglycosylation reactions of denatured lysates with Endo H, the proteins were resolved using 4–12% LDS/NuPAGE in Tris-Bis running, and after transfer of the resolved proteins to membranes the blots were probed with antibodies against the V5 epitope. The masses of Nrf1 proteins based on their electrophoretic migration was estimated to be 120, 95, 85, 55, 46, 36 and 25 kDa. In addition, β-actin was employed as an internal control of protein loading. (**g**) After 6-h transfection with expression plasmids for Nrf1 or its mutants (1.0 μg), *GSTA2-*6 × ARE-Luc (0.5 μg), and pRL-TK (0.1 μg, used to control for transfection efficiency), the COS-1 cells were allowed to recover overnight in 25 mM-glucose medium before being treated with 1 μmol/l MG132 for 18 h, followed by measurement of ARE-driven transcription using the dual luciferase reporter assay. ARE-driven luciferase activity was normalized by the corresponding value of the *Renialla* activity, and then the results were calculated as a fold change (mean ± S.D) of Nrf1 mediated transactivation. Significant increases (^$^p < 0.05 and ^$$^p < 0.001, n = 9) and decreases (*p < 0.05, **p < 0.001, n = 9) are indicated, relative to the background activity measured from pcDNA3-transfected cells (i.e. mediated by endogenous Nrf1 and/or Nrf2).

Most recently, the full-length 95-kDa Nrf1 deglycoprotein has been shown to be proteolytically processed by the proteasome to generate a cleaved 85-kDa protein^37^. Our work has also revealed that the cleaved 85-kDa Nrf1 exhibits a slower electrophoretic mobility than that of the Nrf1^ΔNTD^ mutant (lacking its NTD)^36^, suggesting that generation of the 85-kDa protein may occur by proteolytic processing around the NHB2 region within the NTD. To test this hypothesis, we created a series of expression constructs for Nrf1 mutants within and around NHB2 (Fig. 9a). As expected, when compared with wild-type Nrf1, the abundance of the 85-kDa protein that was stabilized by MG132 was significantly diminished, but not completely abolished, by loss of the entire NHB2 sequence (in Nrf1^Δ81–106^, Fig. 9b) or portions thereof (in Nrf1^Δ81–90^, Nrf1^Δ91–95^ and Nrf1^Δ96–106^, Fig. 9c,d). In all cases the residual 85-kDa protein was retained following 5 μmol/l MG132 treatment, but the proteasome inhibitor completely prevented expression of the low abundance ~80-kDa protein in COS-1 cells transfected with Nrf1^Δ81–90^ and Nrf1^Δ96–106^ (Fig. 9c,d). By contrast, the levels of the 95-kDa Nrf1 deglycoprotein was increased by MG132 treatment, giving a slightly slower migration than that of the untreated mutant proteins. By comparison with wild-type Nrf1, Nrf1^Δ81–106^ and Nrf1^Δ96–106^ yielded lower amounts of the 85-kDa protein (Fig. 9b,d). Further examination revealed that production of the 85-kDa-protein band was blocked in the Nrf1^W103A/L104A^ mutant (in which a tripeptide ^103^AAV^105^ is introduced instead of the original ^103^WLV^105^) and the Nrf1^Δ103–105^ mutant (lacking ^103^WLV^105^) (Fig. 9e). Intriguingly, the abundance of the 85-kDa Nrf1 was partially reduced upon expression of Nrf1^N101A/A102L^ (in which the tripeptide ^100^VAL^102^ is introduced instead of the original ^100^VNA^102^), but was completely repressed by Nrf1^Δ100–102^ (lacking ^100^VNA^102^) (Fig. 9f). It should be noted that the Nrf1^N101A/A102L^ mutation markedly increases aliphaticity (by 76.1%) and hydrophobicity (by 36.4%) of the NHB2 C-terminal ^100^VNAWLV^105^ hexapeptide, whilst these two parameters are decreased by the other three mutants Nrf1^Δ100–102^, Nrf1^Δ103–105^ or Nrf1^W103A/L104A^. Together with previous identification of NHB2 as a topological anchor on the luminal side of the ER membrane^28^, our findings suggest that production of the cleaved 85-kDa Nrf1 is monitored by the local membrane-topology of NHB2. Importantly, expression of the 120-kDa Nrf1 glycoprotein and the 95-kDa Nrf1 deglycoprotein was decreased (rather than abolished) in Nrf1^N101A/A102L^ and Nrf1^Δ100–102^, but not in Nrf1^W103A/L104A^ or Nrf1^Δ103–105^, even in the presence of MG132 (Fig. 9e,f). Collectively, these results indicate that within NHB2, the peptide ^96^PTTEVNAWLVH^106^ is required for either stabilization of Nrf1 or selective processing of the transcription factor.

Intriguingly, removal of the NHB2-flanking CRAC1/2 motif (to yield Nrf1^Δ55–80^) diminished processing necessary to generate the cleaved 85-kDa Nrf1 protein, and also prevented the increase in expression of the 120-kDa Nrf1 glycoprotein that was stimulated by MG132 (Fig. 9d). By contrast, the abundance of the 95-kDa and 85-kDa Nrf1 isoforms was reduced upon expression of the Nrf1^Δ31–80^ mutant (lacking the entire spacer region between NHB1 and NHB2, Fig. 8a,b). MG132 treatment caused a relative increase in the 95-kDa rather than 85-kDa Nrf1, but conversely, the latter 85-kDa protein was increased by deglycosylation to abolish the 120-kDa, whilst no change in the 95-kDa protein was observed (Fig. 9b). Together, these results indicate that the spacer region (aa 31-80) between NHB1 and NHB2 is required for stabilization of Nrf1 and its selective post-translational processing to generate the 120-kDa glycoprotein, the 95-kDa deglycoprotein and the cleaved 85-kDa protein.

Next, we examined whether the relative abundance of the cleaved 85-kDa Nrf1 protein effected gene transcription using a luciferase reporter assay (Fig. 9g). As anticipated, a marked decrease of cleaved 85-kDa protein (in Nrf1^Δ81–106^, Nrf1^Δ81–90^, Nrf1^Δ91–95^ and Nrf1^Δ96–106^, *right panel*) or its complete absence (in Nrf1^W103A/L104A^ and Nrf1^Δ103–105^, *left panel*) significantly diminished MG132-stimulation of reporter gene activity. As an exception, the MG132-induced activity of Nrf1^Δ100–102^ was unaffected by the absence of the cleaved 85-kDa protein was observed (Fig. 9g). Amongst these mutants, only Nrf1^Δ103–105^ exhibited lower basal Nrf1-mediated reporter gene transactivation, whereas Nrf1^W103A/L104A^ exhibited increased basal activity by ~1.5-fold. Furthermore, failure to produce the cleaved 85-kDa protein in Nrf1^N101A/A102L^ (Fig. 9f) caused a ~2-fold increase in its basal activity, but MG132-stimulated reporter activity was unaffected (Fig. 9g). Together with the fact that expression of the 95-kDa Nrf1 deglycoprotein varies in different mutants (Fig. 9b–f), these results demonstrate that Nrf1-target gene transactivation is attributable to differential expression of either the deglycosylated 95-kDa protein or the processed 85-kDa Nrf1 proteins.

### The proteasomal inhibitor MG132 prevents turnover of Nrf1 protein that occurs following its dislocation from the ER lumen into the cyto/nucleoplasm

**Figure 10:**
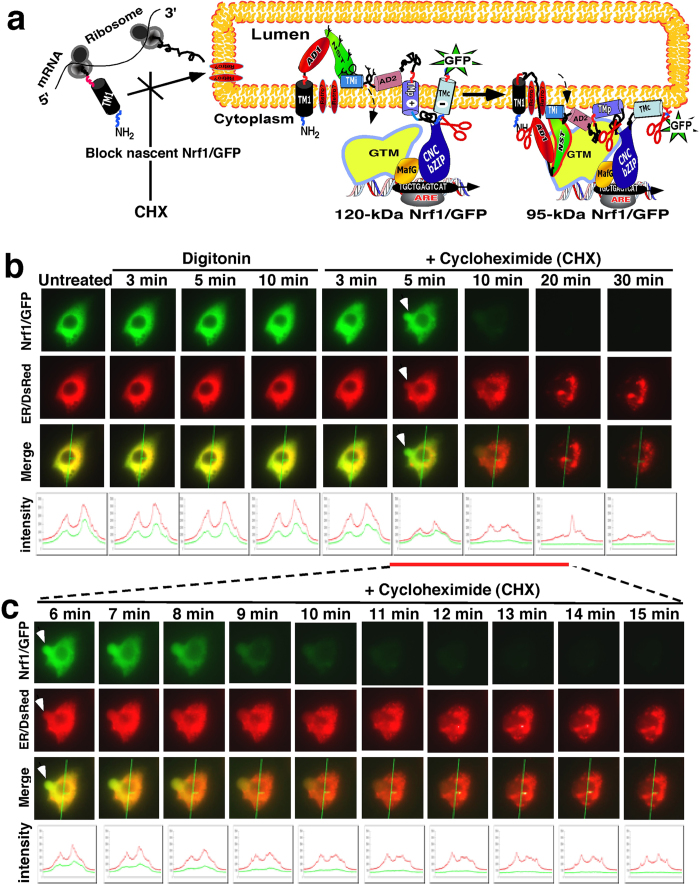
Live-cell imaging of the pre-existing Nrf1/GFP turnover following cycloheximide treatment. (**a**) Distinct membrane-topologies of the 120-kDa and 95-kDa Nrf1 proteins (that have been fused C-terminally by GFP) around and within the ER are schematically shown. The newly synthesized Nrf1 protein from ribosomes can be blocked by cycloheximde (CHX). (**b**) COS-1 cells co-expressing Nrf1/GFP and the ER/DsRed marker were subjected to live-cell imaging combined with the chase experiment. The cells were permeabilized by digitonin (20 μg/ml) for 10 min, before being co-incubated with CHX (50 μg/ml) for additional 30 min. During the course of the experiment, real-time images were acquired using the Leica DMI-6000 microscopy system. The merged images of Nrf1/GFP with ER/DsRed are placed (on *the third row of panels*), whereas changes in the intensity of their signals are shown graphically (*bottom*). The features of arrow-indicated cells are described in the main text. Overall, the images shown are a representative of at least three independent experiments undertaken on separate occasions that were each performed in triplicate (n = 9). (**c**) Additional live-cell imaging of Nrf1/GFP was acquired from 6 to 15 min after co-incubation of CHX with digitonin, as described above.

In our experiments reported in Fig. 1b, ER-localized Nrf1/GFP is dynamically retrotranslocated from the luminal side of the membrane into the cyto/nucleoplasmic side, thereby allowing the fusion protein to be gradually digested by PK. At the same time, PK might also function like cycloheximide (CHX) insofar as it may deplete the newly-synthesized fraction of Nrf1/GFP located on the cytoplasmic side of the ER membrane, because the nascent cytosolic protein ought to be susceptible to proteinase digestion before it is translocated into the ER lumen. To address this possibility, COS-1 cells co-expressing Nrf1/GFP and the ER/DsRed marker were first pre-treated for 10 min with digitonin to pemeabilize cellular membranes before being treated with 50 μg/ml of CHX (to block new protein synthesis, including Nrf1/GFP) for 5–30 min (in the presence of digitonin, Fig. 10a). Upon incubation of the cells with CHX for 5 min, a decrease in the intensity of ether the green Nrf1/GFP or the ER/DsRed marker was observed; both were merged to give rise to an image that revealed the membrane-bound Nrf1/GFP signal did not completely superimpose upon the luminal-resident ER/DsRed signal (Fig. 10b). Subsequently, the remaining Nrf1/GFP signal gradually weakened, and then disappeared, during the CHX incubation time that was extended from 6 min to 11 min (Fig. 10c). By contrast, no obvious changes in the ER/DsRed signal were observed within 6 min to 15 min CHX incubation (Fig. 10c). However, as the time of CHX incubation was extended from 20 min to 30 min, the size of the ER/DsRed image and its signal intensity became smaller and weaker, respectively, than those obtained from 5–15 min CHX-incubated cells, though the remaining ER/DsRed image was retained after incubation with CHX for 60 min (Fig. 10b). In addition, upon exposure of the cells to CHX for 5 min, a ‘hernia-like’ vesicle was observed that protruded from the cytoplasm (indicated by *arrow* in Fig. 10b,c); the convex image that was merged by the predominant Nrf1/GFP signal and relative weak ER/DsRed signal disappeared after 9 min incubation with CHX in the presence of digitonin. These data indicate that in the presence of digitonin CHX quickly inhibits synthesis of new Nrf1/GFP protein, while the pre-existing Nrf1/GFP protein is likely dislocated from the ER into the cytoplasm or nucleoplasm, whereupon it can be rapidly degraded by an ER-associated protease (i.e. the 26S proteasome) within a shorter time interval than required for PK-mediated proteolysis of Nrf1/GFP (Fig. 1b). This observation suggests that the nascent Nrf1/GFP protein may be partially protected against PK digestion before the intact ER-studded ribosomes can be damaged by the proteinase.

**Figure 11:**
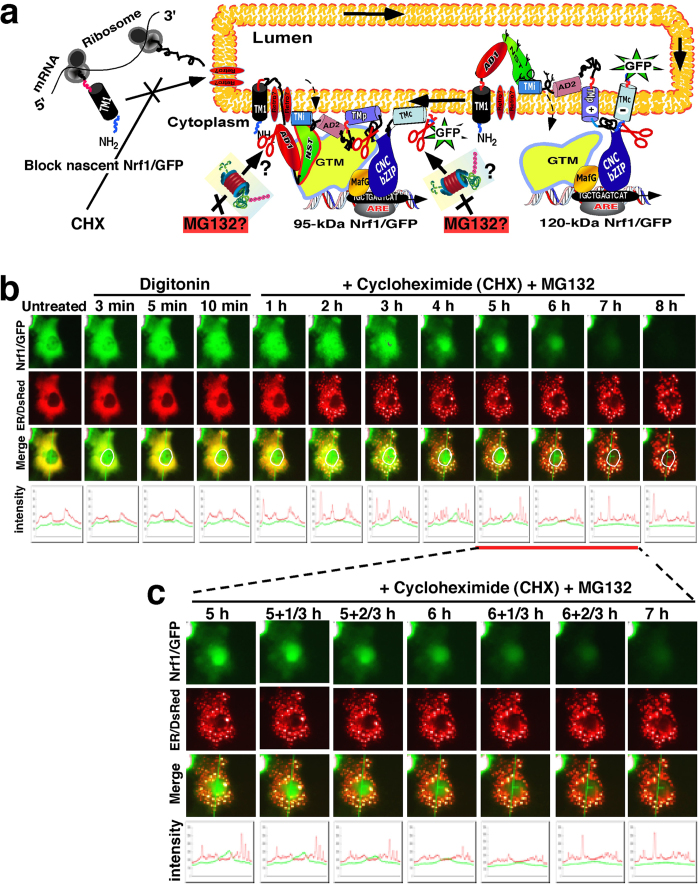
Live-cell imaging of the sensitivity of pre-existing Nrf1/GFP to the proteasome. (**a**) Distinct membrane-topologies of the 120-kDa and 95-kDa Nrf1 proteins around and within the ER are schematically shown. Nrf1 has been fused C-terminally by GFP, which faces the luminal side of membranes during the initial topogenesis. Since Nrf1 is a mobile membrane-protein that entails dynamic topologies, the membrane-topological folding of glycosylated 120-kDa Nrf1/GFP is distinct from that of non-glycosylated/de-glyosylated 95-kDa Nrf1/GFP (also see refs 25,26). Once the C-terminal region of Nrf1 would be repartitioned across the ER membrane into the cyto/nucleoplasmic side, whereupon it is susceptible to proteolytic degradation mediated by the ERAD-dependent proteasome or other cytosolic proteases. The nascent Nrf1 protein from ribosomes can be blocked by cycloheximde (CHX). (**b**) COS-1 cells co-expressing Nrf1/GFP and the ER/DsRed marker were subjected to live-cell imaging combined with the chase experiment. The cells were permeabilized by digitonin (20 μg/ml) for 10 min, before being co-incubated with CHX (50 μg/ml) and MG132 (5 μmol/l) for 8 h. During the course of the experiment, real-time images were acquired using the Leica DMI-6000 microscopy system. The merged images of Nrf1/GFP with ER/DsRed are placed (on *the third row of panels*), whereas changes in the intensity of their signals are shown graphically (*bottom*). The features of arrow-indicated cells are described in the main text. Overall, the images shown are a representative of at least three independent experiments undertaken on separate occasions that were each performed in triplicate (n = 9). (**c**) Additional live-cell imaging of Nrf1/GFP was acquired from 5 to 7 h after co-incubation of CHX and MG132 in the presence of digitonin, as described above.

In order to determine whether (and how long) the pre-existing Nrf1 protein is degraded by the proteasome (or other unidentified proteases), COS-1 cells co-expressing Nrf1/GFP and the ER/DsRed were pre-treated for 10 min with digitonin before being co-treated with 50 μg/ml CHX and 5 μmol/l MG132 for an additional 1–8 h (in the presence of digitonin, Fig. 11a). When compared to untreated cells, no apparent changes in either the Nrf1/GFP or the ER/DsRed marker were found after 10-min incubation with digitonin, but subsequent co-treatment with CHX and MG132 for 1 h to 8 h gave rise to distinct images from Nrf1/GFP and from ER/DsRed, and both signals could not be completely superimposed (Fig. 11b). As the time of co-treatment of the cells with CHX and MG132 was extended from 3 h to 6 h, the cytoplasmic Nrf1/GFP signals became weaker until they gradually disappeared (Fig. 11c), whilst the nuclear Nrf1/GFP signals became relatively enhanced within 3 h co-treatment of CHX and MG132, and then gradually weakened by co-treatment with CHX and MG132 for 4 h to 8 h until it disappeared. However, the remaining ER/DsRed signals appeared to be further unaffected by co-treatment of CHX and MG132 for 3 h to 8 h. Together with the PK-digested data (as shown in Fig. 1), these results indicate that during inhibition of newly-synthesized Nrf1/GFP by CHX, the pre-existing Nrf1/GFP is dislocated from the ER across membranes into the cyto/nucleoplasm, and in these compartments it is susceptible to rapid degradation by the 26S proteasome because the proteasomal inhibitor MG132 significantly inhibits turnover of the ER-associated Nrf1/GFP, but still permits destruction by an MG132-insensitive protease.

## Discussion

In the present study we found that: i) dynamic movement of the Nrf1 polypetide and its potential TAD-adjoining degrons in and out of the ER dictates its post-translational modifications; ii) selective proteolytic processing of Nrf1 yields distinct isoforms that are in turn responsible for transactivation or transrepression of ARE-driven gene expression; and iii) a bidirectional regulatory feedback circuit exists between Nrf1 and the proteasome.

The unique biological function of the integral membrane-bound Nrf1 factor, that is distinct from the soluble Nrf2, is dictated by its topological folding within the ER. Our previous work has revealed that the correct membrane-topological folding of Nrf1 requires the presence of both AD1 and AD2 within its TAD sequences, as well as the NHB1-associated TM1 sequence and the semihydrophobic amphipathic TMi, TMp and TMc (i.e. the C-terminal transmembrane) regions^24^,^25^,^26^. Intriguingly, the local membrane-topology of the TM1-associated NHB1 sequence in Nrf1 appears to resemble that of the TM domains of ATF6 and SREBP1 that span membranes in an N_cyt_/C_lum_ fashion^24^,^25^,^26^. However, the NHB1-adjoining signal sequence in Nrf1 possesses neither Site-1 nor Site-2 protease cleavage sites, and it therefore cannot be processed by a mechanism similar to the regulated intramembrane proteolysis of ATF6 and SREBP1 (refs 24,26). Our present live-cell imaging, together with previous glycosylation mapping analysis, membrane protease protection reactions, and reporter gene assays (refs 24, 25, 26 and this study), demonstrate that the membrane-topovectoral process of Nrf1, by which it moves dynamically into and out of ER membranes, controls the selective post-translational processing that is responsible for producing distinct Nrf1 isoforms.

**Figure 12:**
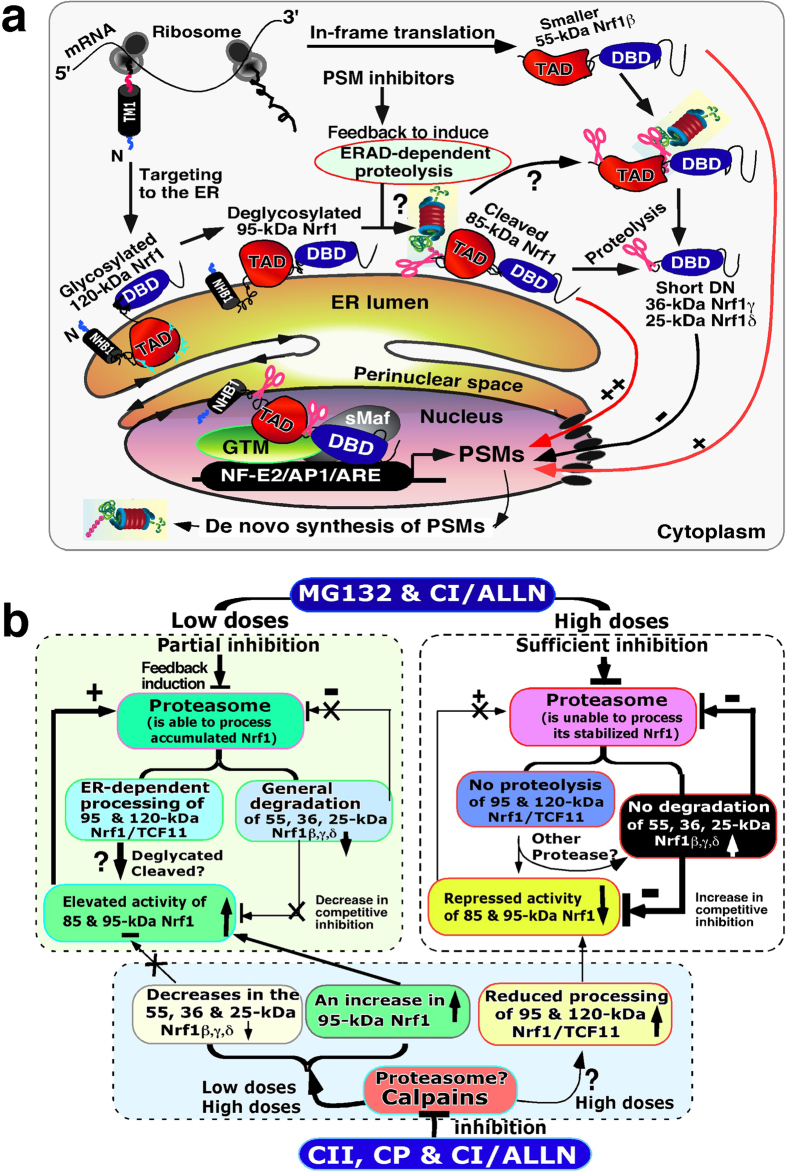
A bidirectional regulatory feedback circuit exists between Nrf1 and the 26S proteasome. (**a**) A model is proposed, based on our findings (refs 23, 24, 25, 26,34,36 and this study) and those of others (refs 2,3,29, 30, 31,37,40), in order to explain the regulatory feedback circuit between Nrf1 and the 26S proteasome. The 120-kDa Nrf1 glycoprotein is retrotranslocated through the p97/VCP-driven pathway from the ER into the nuclo/cytoplasm where it is deglycosylated to yield an active 95-kDa isoform. The latter deglycoprotein is proteolytically processed to generate a cleaved 85-kDa Nrf1 isoform by the 26S proteasome that is activated by proteasome inhibitors through a feedback loop. In turn, transcriptional expression of the 26S proteasome and p97/VCP complexes is regulated by the 95-kDa and/or 85-kDa Nrf1 isoforms before the CNC-bZIP transcription factor is subjected to further proteasome-mediated processing that creates small Nrf1 isoforms of between 55-kDa and 25-kDa. Such small polypeptides may also be produced by in-frame translation of long Nrf1 mRNA transcripts and subsequently further degraded by the proteasome. (**b**) Schematic representation of a bidirectional regulatory feedback circuit exists between Nrf1 and the 26S proteasome. The transcriptional expression of 26S proteasomal subunits (e.g. PMSA4 and PMSB6) is regulated predominantly by Nrf1 (see refs 2,3,29, 30, 31,37,40). In turn, different doses of the proteasomal inhibitors MG132 and CI/ALLN exert opposing effects on the activity of Nrf1 to mediate expression of genes encoding proteasomal subunits. Specifically, at low doses, MG132 and CI/ALLN increase activity of Nrf1-target proteasomal subunit gene expressions by increasing the levels of the active 95- and/or 85-kDa Nrf1 isoforms, whereas high doses of MG132 and CI/ALLN inhibit Nrf1-mediated transcription by increasing the levels of the 36-kDa Nrf1γ and 25-kDa Nrf1δ dominant-negative isoforms. However, the detailed rational mechanism(s) of this bi-phasic response remains to be determined. In addition, further mechanistic study is warranted to identify which proteases enable the endoproteolytic processing of either 120-kDa or 95-kDa Nrf1 proteins to yield the cleaved 85-kDa isoform.

Nrf1 is synthesized as a non-glycosylated 95-kDa polypeptide by ribosomes associated with the ER (Fig. 12a). If the non-glycosylated 95-kDa protein is retained in the cyto/nucloplasm (as principally occurs in tunicamycin-treated cells), it is susceptible to proteolytic degradation and thus exhibits about 75% of the transactivation activity of the wild-type Nrf1 factor treated with vehicle^8^,^26^. During the initial membrane-topogenesis of Nrf1, the nascent 95-kDa polypeptide is co-translationally integrated within and around the ER through its membrane-associated regions, whilst its TAD sequences (including AD1, NST, AD2 and SR) and its DNA-binding CNC-bZIP domain are partitioned into the luminal and cyto/nucloplasmic sides of membranes, respectively. The NST domain of Nrf1 is glycosylated in the ER so that it exists as a 120-kDa glycoprotein, which is likely to be inactive because its TAD sequences are buried within the lumen and therefore cannot mediate transactivation of nuclear target genes. Upon deletion of the N-terminal aa 2–10, the resulting Nrf1^Δ2–10^ mutant is over-expressed as a major 120-kDa glycoprotein, which appears to be further processed to yield three minor polypeptides of 85-kDa, 55-kDa and 36-kDa; together, they exhibit approximately 30% of wild-type Nrf1 transactivation activity (ref. 24).

Induction of Nrf1-target genes requires the luminal AD1, AD2 and the NST glycodomain of the 120-kDa glycoprotein to be repartitioned out of ER, so that they can be retrotranslocated and/or dislocated from the ER lumen across membranes into the cyto/nucleoplasmic subcellular compartments, whereupon Nrf1 may be deglycosylated to generate an active 95-kDa transcription factor (Fig. 12a). Following stimulation of Nrf1 by glucose deprivation in medium containing 2.5 mM glucose, the non-glycosylated/deglycosylated 95-kDa protein accumulates and is accompanied by an approximate 3.8-fold increase in Nrf1 activity when compared with its activity under control conditions in which cells were grown in medium containing 25 mM glucose (ref. 25 and this study). However, it remains to be determined which ER-to-cytosol retrotranslocon-competent proteins, such as Derlins and the Sec61 complex (refs 38,39), are responsible for the repartitioning of Nrf1 from the ER into the nucleus. Furthermore, it also remains to be established how the putative topovectorial processing of Nrf1 is stimulated by glucose deprivation.

Both the membrane-bound 120-kDa and 95-kDa Nrf1 proteins, besides being respectively glycosylated and presumably deglycosylated (and partially newly-synthesized non-glycosylated protein), are subject to selective proteolytic processing to yield a cleaved 85-kDa isoform and other smaller polypeptides of between 60-kDa and 25-kDa (Fig. 12a). We deduce from comparisons of the distinct behaviour of Nrf1 mutants lacking varying lengths of the 170 N-terminal residues (refs 24,28), that the cleaved 85-kDa Nrf1 form is a potential transcriptional activator, because portions of its NTD (aa 1–124) that negatively regulate the CNC-bZIP factor have been removed, but the molecular details of the mechanism(s) has not as yet been identified so far. Furthermore, the cleaved 85-kDa Nrf1 appears to be over-expressed in the case of Nrf1^Δ31–50^ (lacking a putative hinge region adjacent to the NHB1 signal anchor sequence)^28^, but formation of this isoform is partially prevented by deletion of either aa 31–80 (containing a putative Hrd1-binding site^30^) or aa 55–80 (covering CRAC1/2)^28^; this is further confirmed in the present study. Notably, none of the four fusion proteins NTD/GFPx2, NTD/Nrf2, N156/Nrf2 and N170/Nrf2 (within the latter two chimaera, N156 and N170 indicate the N-terminal 156 or 170 aa of Nrf1, respectively) seem to be expressed as equivalents to the cleaved 85-kDa polypeptide^24^,^28^. Collectively, these findings suggest that the NHB1-directed membrane-topology of Nrf1 dictates selective proteolytic processing of the NTD, particularly aa 31–80, possibly through an ER-resident protease-mediated mechanism.

Recently, Sha and Goldberg have shown that the cleaved 85-kDa Nrf1 is generated through proteasome-mediated processing of the full-length 95-kDa Nrf1 deglycoprotein^37^. However, our cycloheximide-treatment experiments^26^ revealed that 5 μmol/l MG132 increases the half-life of the 85-, 95- and 120-kDa Nrf1 proteins (for ~27, 93 and 151 min, respectively) approximately 33-, 5.0- and 2.3-fold, suggesting that the cleaved 85-kDa protein is further degraded by the cyto/nucleoplasmic 26S proteasome, whereas the ER-associated 95-kDa and 120-kDa Nrf1 proteins are proteolytically processed by an MG132-insensitive pathway to yield the cleaved 85-kDa and smaller isoforms. Intriguingly, we have also found that the cleaved 85-kDa Nrf1 exhibits a relatively slower electrophoretic mobility than that of the Nrf1^ΔNTD^ protein that lacks its NTD^36^, suggesting that processing of Nrf1 to generate the 85-kDa protein occurs within its NHB2-adjoning regions. This conclusion is not consistent with our failure to observe a polypeptide from NTD/GFPx2, NTD/Nrf2, N156/Nrf2 or N170/Nrf2 that is equivalent to the 85-kDa protein^8^,^24^,^28^. Together with previous membrane protection assay data (showing that NHB2 acts as an ER luminal-resident anchor, and the existence of other topogons within the NHB2-conneting domains)^8^,^25^,^28^, we postulate that the post-translational processing of Nrf1 is monitored by membrane-topological repartitioning of its NHB2-connecting regions (i.e. AD1, NST and AD2, which do not have equivalents in Nrf2) from the luminial side of the ER membranes into the cyto/nucleoplasmic side (Fig. 12a), whereupon Nrf1 is targeted for deglycosylation, ubiquitination, and selective proteolysis by the ER-associated proteasome (or other unidentified proteases) to generate the cleaved 85-kDa and other smaller isoforms. This proposal is supported by our finding that generation of the cleaved 85-kDa Nrf1 is significantly blunted by mutants lacking portions of NHB2, particularly the C-terminal ^100^VNAWLV^105^ hexapeptide. By contrast, none of the NHB2 mutants block formation of the 95-kDa Nrf1 deglycoprotein, although its abundance is variable. Furthermore, the NHB1 deficient mutants (e.g. Nrf1^Δ2–36^ and Nrf1^Δ11–23^) give rise to an intact major cytosolic non-glycosylated Nrf1 protein, with neither a cleaved 85-kDa protein nor a deglycosylated 95-kDa isoform being recovered (see refs 8,24,31 and data not shown). Additional treatment of cells with tunicamycin (to block N-linked glycosylation) allows wild-type Nrf1 to be expressed as a major full-length non-glycosylated protein^8^,^24^,^26^,^31^. Together, these findings demonstrate that the post-translational processing of wild-type Nrf1 occurs through glycosylation, deglycosylation and selective proteolysis within and around the ER, but it is not known why Nrf1 is processed through the organelle.

The topovectorial processing of Nrf1 is modulated by the VCP/p97-driven retrotanslocation and Hrd1-dependent ubiquitin proteasomal pathways^2^,^30^,^37^,^40^. Thus Nrf1 is positively regulated by VCP/p97-mediated ER-to-cytosolic extraction, and is then negatively regulated by Hrd1-dependent ER-associated degradation (ERAD) (see Fig. S7). This proposed multi-tiered control of Nrf1 is supported by the finding that it is activated in response to lower doses of proteosomal inhibitors that reduce degradation of both the active 95-kDa and 85-kDa Nrf1 isoforms, but is also repressed by higher doses of proteosomal inhibitors that diminish degradation of its smaller dominant-negative isoforms (i.e. 36-kDa and 25-kDa) (see a proposed model in Fig. 12b, & ref. 37). Recently, generation of the cleaved 85-kDa Nrf1 arising from its 95-kDa deglycoprotein has been shown to be bi-directionally regulated by the proteasome, which is dependent on distinct doses of proteosomal inhibitors (i.e. lower doses increases the 85-kDa protein abundance, whereas higher doses inhibit its production^37^). However, increases in the abundance of both the cleaved 85-kDa Nrf1 and the 95-kDa non-glycosylated and/or deglycosylated protein (ref. 26 and this study) indicate that the proteosome-mediated processing of Nrf1 is unlikely to occur at the higher doses of the MG132 proteosomal inhibitor through a mechanism similar to the endoproteolytic cleavage of the fully-synthesized p105 precursor of NF-κB. The proteasomal cleavage enables selective proteolytic degradation of the p105 C-terminus to yield the active p50 NF-κB factor in a ubiquitin-independent manner^41^. The mechanism underlying the endoproteolytic processing of the full-length Nrf1 (i.e. 120-kDa glycoprotein and 95-kDa non-glycosylated proteins) by an unidentified proteosomal inhibitor-insensitive protease to generate the cleaved 85-kDa and/or other smaller isoforms remains to be studied. In addition, another unstable ~70-kDa N-terminally-truncated Nrf1 protein is detected on few occasions, but its production is likely to be completely inhibited by MG132 (Fig. 7c and S3C).

Further proteolytic processing of the 120-, 95- and 85-kDa Nrf1 proteins generates several TAD-deficient isoforms, such as those of approximately 55-kDa, 36-kDa and 25-kDa (designated Nrf1β/LCR-F1, Nrf1γ and Nrf1δ, respectively in Fig. S4)^8^,^26^,^42^,^43^. In the case of 55-kDa LCR-F1/Nrf1β it arises primarily from internal translation codons located between Met^289^ and Met^297^ within the longer transcripts^44^,^45^,^46^,^47^, By comparison with full-length Nrf1, the 55-kDa Nrf1β/LCR-F1 lacks both the NTD and the essential AD1 region, but still contains the AD2, NST and SR domains that contribute to transactivation activity. Thus, LCR-F1/Nrf1β is a soluble nuclear activator with weak transactivation activity^8^,^35^,^36^,^44^,^48^. However, one research group has reported that LCR-F1/Nrf1β is a dominant-negative inhibitor of ARE-driven gene transactivation by the full-length Nrf1 and/or Nrf2 factors^47^. This disparity suggests that LCR-F1/Nrf1β may be unstable and is rapidly degraded to yield isoforms of between 46-kDa and 25-kDa (see refs 8,36,42,48).

The 36-kDa Nrf1γ and the 25-kDa Nrf1δ isoforms, which arise from in-frame translation and/or selective proteolysis, are two *bona fide* dominant-negative isoforms because they both lack potential TAD regions (i.e. AD1, AD2, NST and SR) (refs 8,35,42 and this study). This conclusion is based on the fact that when 36-kDa Nrf1γ and 25-kDa Nrf1δ, are over-expressed, they can competitively interfere with the function of intact wild-type full-length Nrf1 and/or Nrf2 (ref. 36), thereby inhibiting induction of target genes. Furthermore, generation of the 25-kDa Nrf1δ isoform was, to a certain extent, prevented by expression of either Nrf1β^M523/548L^ or Nrf1^M523/548L^ (in which Met^523^ and Met^548^ are both replaced with leucine, Fig. S5), which exhibit an increased activity when compared with the corresponding wild-type factor forms^26^. By contrast, formation of 36-kDa Nrf1γ is diminished by deletion of putative AD2-adjoining degrons, the DSGLS-containing SDS1 motif (aa 441–455) and/or the PEST2 sequence (aa 456–519), resulting in significant increases in its activity. Therefore, we suggest that these two degrons suppress Nrf1 activity through selective proteolytic processing of its longer proteins into small dominant-negative forms. However, the failure to generate ~36-kDa Nrf1γ from the Nrf1^Δ454–488^ mutant (lacking SR region as a regulated TAD) does not cause an increase in both its basal and inducible activity to mediate ARE-driven gene expression. Conversely, the presence of ~36-kDa Nrf1γ does not block significant increases in both the basal transactivation activity of the Nrf1^ΔDSGLS^ mutant (lacking DSGLS degron) and its stimulation by glucose deprivation. These intriguing observations suggest that the ~36-kDa Nrf1γ is unlikely to act as a determinant of the transrepressor activity to compete against the mutants Nrf1^Δ454–488^ and Nrf1^ΔDSGLS^. In addition, it should be noted that variations in the yield of the shorter Nrf1 isoforms between 55-kDa and 25-kDa and their selection from the proteolytic processing of the larger proteins (i.e. between 120-kDa and 85-kDa) also appear to be attributable to distinct cellular states within different experimental settings (e.g. passages and confluences), that influence cellular biological process and functional response to proteolytic cues.

Collectively, our findings demonstrate that the function of Nrf1 is influenced by selective topovectorial processing mechanisms. The repression of Nrf1 is also monitored by its several potential degrons that target it for proteasome- and/or calpain-mediated proteolysis. Neither the PEST1 sequence nor the Neh2L subdomain within AD1 act as *bona fide* degrons for Nrf1, at least when they are buried in the ER lumen, but conversely contribute to the stability of the 120-kDa glycoprotein. By contrast, both the AD2-adjoining DSGLS motif and the PEST2 sequence, that overlap the Neh6L domain, negatively regulate Nrf1 by allowing proteasome- and/or calpain-mediated proteolytic processing to produce Nrf1β/LCR-F1, Nrf1γ and Nrf1δ. Moreover, the CPD degron (situated immediately to the Neh5L within AD1) and the DSGLS motif in Nrf1 are reported to be recognized by FBW7 and β-TrCP, respectively, which target the 95-kDa protein for cullin-1 directed proteasomal degradation^29^,^30^. Overall, selective proteolytic processing of Nrf1 is dependent on the positioning of the putative cleavage sites around and within membranes, but detailed mechanisms remain to be determined.

In the present study we have also shown that proteasome inhibitors have dual opposing effects on Nrf1 and that in turn, Nrf1 regulates expression of the 26S proteasomal subunits. This conclusion is also supported by a recent publication by Sha and Goldberg^37^ (while our work was under review for near 2 years). Together with the findings of others^2^,^3^,^21^, it appears that Nrf1 controls the ‘bounce-back’ response to proteasome inhibitors insofar as it allows induction of 26S proteasomal subunits as an adaptive recovery response to inhibition of the proteasome^2^,^3^,^21^. We therefore propose that a bidirectional regulatory feedback circuit exists between Nrf1 and the proteasome (Fig. 12b), and envisage that the regulatory feedback enables Nrf1 to maintain cellular homeostasis and organ integrity. Hence, it is reasonable to hypothesize that during normal development and growth, organ integrity is fine-tuned by a steady-state balance between the selective proteolytic processing of Nrf1 by the 26S proteasome-dependent (and -independent machineries) and the transcriptional activity of Nrf1 to regulate the expression of genes encoding proteasome subunits.

Notably, molecular cloning of Nrf1 and its long form TCF11 reveals that in human these two polypeptides comprise 742 and 772 aa, respectively; Nrf1 arises from alternatively splicing of the full-length transcript to delete Exon 4 encoding aa 242–271of TCF11 (refs 49,50). Almost equal amounts of Nrf1 and TCF11 are detected to be co-expressed in some normal human cell lines, whilst human cancer cell lines express predominantly Nrf1 rather than TCF11 (data not shown). In mouse tissues and cell lines, only Nrf1 (of 741 aa) is expressed, with not TCF11 (refs 35,51). ARE-driven luciferase assays showed that Nrf1 and TCF11 have similar abilities to mediate basal expression of an ARE-driven reporter gene; the transactivation activity is positively regulated by AD1 (ref. 46), even though AD1 of Nrf1 lacks a putative leucine-rich nuclear export signal (^250^A*L*S*L*EEC*L*R*LL*EA^262^) within the aa 242–271 region (^242^VPSGEDQTALSLEECLRLLEATCPFGENAE^271^) of TCF11 (ref. 52). This acidic-hydrophobic region was designated as a Neh4-like (Neh4L) subdomain of AD1 because it is highly conserved with the Neh4 transactivation domain (^105^VAHIPKQDA*L*Y*F*EDC*M*Q*LL*AETFPFVDDHE^134^) of Nrf2 (see Fig. S1B). Intriguingly, Krugur and colleagues have reported that human TCF11 plays a major role in regulating transcriptional expression of 26S proteasomal subunits in the ‘bounce-back’ response to proteasome inhibitors, whilst human Nrf1 was thought to make a minor contribution in this response^2^. However, Nrf1 rather than Nrf2 in model mouse (lacking TCF11) has been reported to be essential for basal and inducible expression of 26S proteasomal subunit genes^3^,^29^,^53^,^54^. Although the different contributions of Nrf1, TCF11 and Nrf2 to gene regulation remain elusive, we surmise that when differential regulation of target genes by Nrf1 and TCF11 is required to maintain cellular homeostasis (i.e. proteostasis), these two ER-associated CNC-bZIP proteins will be selectively subjected to the membrane-topological processing to yield distinct functional isoforms. Therefore, it is plausible that topovectorial processing of Nrf1 and TCF11 occurs likely *via* similar ER-to-cyto/nucleoplasmic mechanisms as described (refs 2,26,36,37,40 and herein). However, the presence of ^250^*A*LS*L*EE*CL*R*LL*EAT*C*PF*G*^268^ (which is predicted to be folded into an amphipathic helix with a redox-sensitive hydrophobic side and another net acidic side) within the Neh4L region of TCF11, rather than Nrf1, facilitates retrotranslocation of the former protein from the ER luminal side across membranes into the cytoplasmic side and its ensuing dislocation into the nucleus before transactivating target genes. It is postulated that the topovectorial processing of TCF11, but not Nrf1, might be regulated by certain modification of its cysteine residues within ^250^*A*LS*L*EE*CL*R*LL*EAT*C*PF*G*^268^ in response to a putative redox signalling sensor in the proximity of ER-to-cyto/nucleoplasmic compartments.

**Figure 13:**
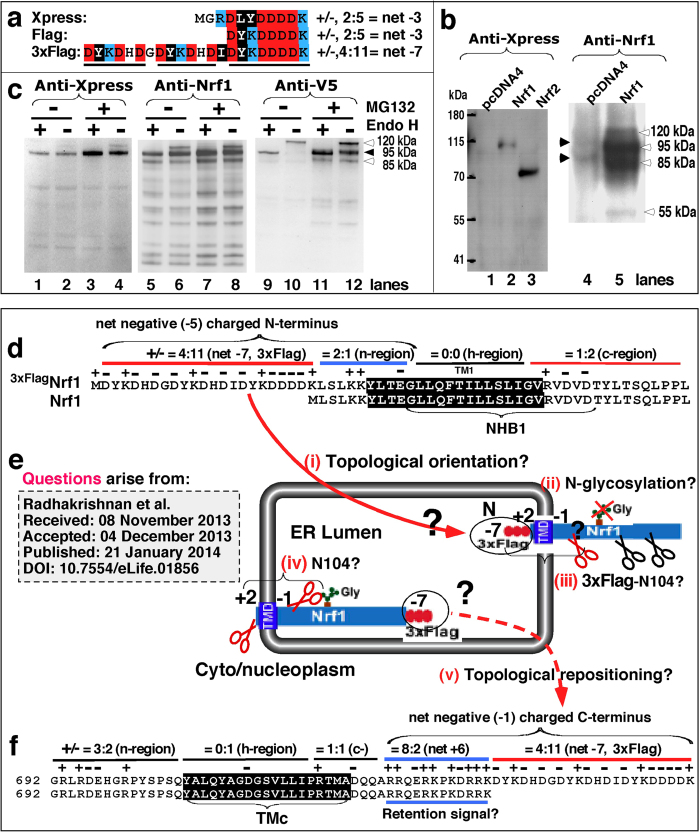
The membrane-topology of ectopic Nrf1 can be dictated by the negative charged 3xFlag or Xpress epitopes. (**a**) Amino acid alignment reveals that the net negative charged Xpress epitope is highly conserved with Flag and 3xFlag. Acidic and basic amino acids are placed in the red and blue backgrounds, respectively, whereas the hydrophobic amino acids are on a black background. (**b**) Total lysates from COS-1 cells that had been transfected with pcDNA4/HisMax-based expression constructs for Nrf1, Nrf2, or an empty vector (i.e. pcDNA4), were separated by 7% NuPAGE in a Tris-Acetate running buffer, followed by immunoblotting with an Xpress antibody. The immunoblotted nitrocellulose membrane was then re-probed with an additional antibody against Nrf1 (*right panel*). (**c**) Total lysates from COS-1 cells expressing N-terminally Xpress-tagged Nrf1 or C-terminally V5-tagged Nrf1, which had been treated for 4 h with proteasomal inhibitor MG132, were subjected to Endo H-catalyzed deglycosylation reactions, before being resolved using 4–12% LDS/NuPAGE in Tris-Bis running buffer, and then immunoblotted using Xpress or V5 antibodies. The anti-Xpress blotted nitrocellulose membrane was re-probed with Nrf1 antibody (*middle panel*). (**d**) Amino acid alignment of the TM1-associated regions from ^3xFlag^Nrf1 and the intact Nrf1. The charge difference between the 3xFlagged n-region (net -5) and the c-region (net -1) directs the TM1 region of ^3xFlag^Nrf1 to adopt an N_lum_/C_cyt_ orientation within the ER membrane. By contrast, the net positive n-region (+2) enables the TM1 region within intact Nrf1 to adopt an N_cyt_/C_lum_ orientation. (**e**) Several questions about Nrf1 processing that have arisen from recently published work^40^ have been re-interpreted in the main text, using current knowledge of membrane-topology^55^,^56^ as a guiding principle. Notably, the strongly negative charged 3xFlag dictates the folding of ^3xFlag^Nrf1 and Nrf1^3xFlag^ to adopt non-native membrane-topologies, which make distinctive contributions to the post-translational processing of Nrf1. (**f**) Amino acid alignment of the TMc-associated regions from Nrf1^3xFlag^ and the intact Nrf1. The original positive (+6) C-terminus of Nrf1 enables it to be topologically repositioned into the cytoplasmic side of membranes, whereas the net negative (-1) 3xFlagged C-terminus of Nrf1^3xFlag^ favours location with the ER lumen.

While our work was under review, Radhakrishman *et al.*^40^ reported that Nrf1 is dislocated through p97/VCP-directed retrotranslocation from the ER into the cytoplasm, where it is endoproteolytically cleaved by an unidentified protease to remove its N-terminal 104-aa residues. The model they proposed does not exclude regulation of Nrf1 by its membrane-topology (in our proposed model), because the putative proteolytic cleavage is one of several successive molecular events that occur during membrane-topogenic processing of Nrf1. Importantly, some of the data reported by Radhakrishman *et al.*^40^ merit discussion. Firstly, they found opposing results from membrane protease protection assays of Nrf1 in HEK293 cells depending on whether the CNC-bZIP factor was N-terminally or C-terminally 3xFlag tagged (ref. 40). This suggests that the membrane-topologies of ^3xFlag^Nrf1 or Nrf1^3xFlag^ are dictated by the strongly negative 3xFlag (-7) peptide sequence attached to the N- and C-terminal ends of Nrf1, respectively (Fig. 13d–f). According to membrane-topology dogma^55^,^56^, the orientation of the intact membrane-bound Nrf1 (refs 24, 25, 26,28) is determined by the positive-inside and charge difference rules, and the hydrophobic gradient along membrane (see Fig. 1a). It is therefore possible that the net negative charged (-7) 3xFlag enables the TM1 region of ^3xFlag^Nrf1 to adopt an orientation of N_lum_/C_cyt_ (i.e. its N- and C-termini face the ER lumen and cytoplasm, respectively), whilst the TM1 region of Nrf1^3xFlag^ orientates in an N_cyt_/C_lum_ fashion (Fig. 13e). Secondly, if the TM1 region of ^3xFlag^Nrf1 is integrated in the N_lum_/C_cyt_ orientation and anchored within the ER membrane, its NST domain might be positioned on the cytoplasmic side, thereby preventing its glycosylation. This interpretation is supported by our finding that N-terminally Xpress-tagged Nrf1 (in which Xpress is highly conserved with Flag, in Fig. 13a) migrated electrophoretically as a major 95-kDa protein and does not appear to be glycosylated (Fig. 13c). Once the NST-connecting C-terminal portions of ^3xFlag^Nrf1 was partitioned on the cytoplasmic side, it would be not protected by the ER membranes against protease digestion, as reported by Radhakrishnan *et al.*^40^. Thirdly, if the TM1 region of Nrf1^3xFlag^ is folded in an N_cyt_/C_lum_ orientation, its NST domain would be translocated into the ER lumen, where it can be glycosylated to generate a 120-kDa glycoprotein. However, the strongly negative charged 3xFlag may make it more likely that the C-terminal portion of Nrf1^3xFlag^ will reside on the ER luminal side such that Nrf1^3xFlag^ is partially protected by membranes against protease digestion as reported by Radhakrishnan *et al.*^40^. Fourthly, the authors indicated that a mix of amino acid sequences ^104^LVHRD^108^ and ^105^VHRD^108^ was also generated during sample processing of Nrf1^3xFlag^, but the putative cleaved N-terminal 104 aa fragment of ~12 kDa was not shown by electrophoresis of Nrf1^3xFlag^ or ^3xFlag^Nrf1. Further evidence that has been presented by Sha and Goldberg^37^ (in their supplemental Figure S4) demonstrates that none of the exact proteasomal cleavage sites existing within the N-terminal domain of Nrf1-HA were determined by the liquid chromatography-mass spectrometry (LC-MS)/MS although some proteasome activity is required for the processing of the CNC-bZIP factor to release a putative cleaved protein from the ER into the nucleus.

Herein, we have also provided evidence that generation of the cleaved 85-kDa Nrf1 is prevented by mutants lacking the C-terminal hexapeptide ^100^VNAWLV^105^ of NHB2, and is also significantly diminished, but not completely abolished, by loss of the entire NHB2 sequence. By contrast, generation of the non-glycosylated and/or deglycosylated 95-kDa Nrf1 is not abolished by loss of either the NHB2 sequence or various portions of the NHB2 and its flanking sequences between NHB1 and NHB2 (Fig. 9). However, further mechanistic studies are warranted to explain how proteolytic processing of Nrf1 by ER-associated proteases generates the cleaved 85-kDa protein, though it should be recognised that Sha and Goldberg^37^ showed that the proteasome mediates the proteolytic processing of Nrf1.

In summary, distinct membrane-topogenic events enable Nrf1 to be selectively processed within different temporo-spatial subcellular locations (from the ER into the cyto/nucleoplasm), where it is specifically modified in a variety of ways (i.e. glycosylation, deglycosylation, phosphorylation, ubiquitination, degradation and proteolytic cleavage) to yield multiple distinct isoforms (i.e. 120-kDa, 95-kDa, 85-kDa, 55-kDa, 36-kDa and 25-kDa). Collectively, the Nrf1 forms finely tune basal and inducible expression of cognate genes that provide protection against distinct cellular stressors, particularly those derived from the ER. Amongst these isoforms, expression of the cleaved 85-kDa Nrf1 protein is blocked by loss of the ER luminal-anchoring NHB2-adjoining sequences, whereas production of the dominant-negative 36-kDa Nrf1γ protein is abolished by removal of the DSGLS-containing SDS1 and/or its flanking PEST2 sequences.

## Materials and Methods

### Chemicals and antibodies

All chemicals were of the highest quality commercially available, with proteosome and calpain inhibitors being purchased from Sigma-Aldrich. Digitonin, endoglycosidase (Endo) H and proteinase K (PK) were obtained from New England Biolabs. Two mouse monoclonal antibodies against the V5 or Xpress epitope were from Invitrogen Ltd. Antisera against Nrf1were produced in rabbits using a polypeptide covering aa 292–741.

### Expression constructs

Expression constructs for full-length mouse Nrf1 have been described previously^8^,^23^. Mutants were created by PCR-directed point or deletion mutagenesis within the TADs, SR/PEST2 or Neh6L of Nrf1, as described previously^57^. The fusion proteins Nrf1/GFP and N275/GFP, as well as DsRed-GFP, were engineered by ligating the cDNA sequence encoding the full-length Nrf1 and its N-terminal 275 amino acids (N275, covering the entire NTD and most of AD1) with the cDNAs for GFP, as described previously^8^. To express N-terminally Xpress-tagged Nrf1, its encoding cDNA sequence was cloned in the KpnI/XbaI site of pcDNA4/HisMax. The fidelity of all cDNA products was confirmed by sequencing.

### Cell culture, chemical treatment, transfection, and reporter gene assays

Unless otherwise indicated, monkey kidney COS-1 cells (3 × 10^5^) were seeded in 6-well plates and grown for 24 h in DMEM containing 25 mM glucose and 10% FBS. After reaching 70% confluence, the cells were transfected with a Lipofectamine 2000 (Invitrogen) mixture that contained an expression construct for wild-type Nrf1 or a mutant protein, together with *P*_*SV40*_*GSTA2*-6 × ARE-Luc, which contains six copies of the core ARE consensus sequence from rat *GSTA2* (refs 8,23,58), 3 × *PMSA4*-ARE-Luc, which contains three copies of the core ARE from the promoter of *PSMA4* (refs 3,30), or pCAT1500, which comprises an ARE-containing 1500-bp sequence from the *PMSB6* promoter^2^, along with pcDNA4/HisMax/*lacZ* encoding β-galactosidase (β-gal) that was used as a control for transfection efficiency. In addition, mutant versions of these reporter genes that lacked the ARE sequence were used as negative controls. Luciferase or chloramphenicol acetyltransferase (CAT) activity was measured approximately 36 h after transfection of cells, which were then treated for 18 h with various doses of proteasome and/or calpain inhibitors. The basal and stimulated ARE-driven reporter gene activity obtained following transfection with an expression vector for Nrf1 (or its mutants) was calculated as a ratio of its value against the background activity (i.e. the luciferase acitivity obtained following co-transfection of an empty pcDNA3.1/V5 His B vector and an ARE-driven reporter after subtraction of the non-specific value from cotransfecting an empty pcDNA3.1/V5 His B vector and a non-ARE-containing Luc plasmid). Subsequently, the basal activity of full-length wild-type Nrf1 was given the value of 1.0, and other data were calculated as a fold change (mean ± S.D) relative to this value. The data points presented each represent at least 3 independent experiments undertaken on separate occasions that were each performed in triplicate. Differences in their transcriptional activity were subjected to statistical analysis.

### Cell viability assays after treatment with proteasomal and calpain inhibitors

COS-1 cells (5 × 10^3^) were seeded in each well of a 96-well plate and cultured overnight in DMEM containing 25 mM glucose and 10% FBS. After reaching approximately 80% confluence, the cells were treated for 18 h with various doses of MG132, CI/ALLN, CII and CP in DMEM containing 5.5 mM or 25 mM glucose. Subsequently, the cells were washed with PBS and then incubated for additional 2 h with 100 μl of a mix of CellTiter 96® Aqueous One solution (MTS, from Promega) and DMEM containing 25 mM glucose and 10% FBS (i.e. V_MTS_:V_DMEM_ = 1:10), before the absorbance (λ = 490 nm) was measured by the Microplate Rader Model 680 (from Bio-Rad) to calculate cell viability. The data presented each represent at least three independent experiments undertaken on separate occasions that were each performed in quadruplicate. The significance of differences in cell viability, relative to untreated control, was subjected to statistical analysis.

### Live-cell imaging combined with *in vivo* membrane protease protection assays

COS-1 cells (1 × 10^6^) were seeded in 35-mm dishes and cultured overnight in 25 mM-glucose medium. The cells were then cotransfected for 6 h with 3 μg of DNA of each expression construct for Nrf1/GFP or N275/GFP^26^, together with 0.5 μg of DNA encoding ER/DsRed, a luminal-resident protein marker of ER^23^,^24^. Subsequently, the cells were allowed to recover from transfection for 16 h, and then subjected to *in vivo* membrane protease protection assays, along with live cell imaging to determine movement of the luminal-resident protein out of ER into the cytoplasmic subcellular compartments, as reported elsewhere^59^,^60^. Briefly, the plasma membranes of COS-1 cells were permeabilized by digitonin (20 μg/ml) for 10 min. Thereafter, the cells were subjected to *in vivo* membrane protection reactions against digestion by PK (50 μg/ml) for between 5 min and 30 min before addition of 0.1% (v/v) TX). To determine whether pre-existing Nrf1 is degraded by the proteasome, the permeabilized cells expressing Nrf1/GFP were treated with cycloheximide (50 μg/ml) alone or in combination with the proteasomal inhibitor MG132 (5 μmol/l). Within the experimental time, live-cell images were acquired at 1 min intervals under a 40× objective lens mounted on Leica DMI 6000 green and red fluorescence microscopes equipped with a high-sensitivity Hamamatsh ORCA-ER camera, cell environment control units (at 37 °C in 5% CO_2_ culture conditions), and a definitive focus module. Relative fluorescence units were measured with Simulator SP5 Multi-Detection system for GFP with 488-nm excitation and 507-nm emission, and for DsRed with 570-nm excitation and 650-nm emission.

### Deglycosylation reactions and western blotting

COS-1 cells (3 × 10^5^) that had been transfected for an indicated time (e.g. 6, 12, or 18 h with Nrf1 and its mutants (1.2 ~ 2 μg of cDNA) before being recovered for additional 18 or 24 h in a fresh complete medium. The total lysates were then subjected to *in vitro* deglycosylation reactions with 500 units of Endo H, followed by protein seperation by LDS/NuPAGE containing indicated concentrations of polyacrylamide running in the indicated buffers, prior to western blotting with the V5 antibody or others. Subsequently, the immunoblotted membranes were developed to enhanced chemiluminescence (ECL) or SuperSignal West Femto maximum sensitivity substrate (Thermo) and then was visualized by the exposure to autoradiographic X-ray film (which was performed in Scotland) or by the automatic imaging system (i.e. VersaDoC^TM^-4000MP from Bio-Rad) (which was carried out in China Mainland), respectively. On some occasions, the nitrocellulose membranes that had already been blotted with an antibody were washed for 30 min with stripping buffer before being re-probed with an additional primary antibody against β-Actin; this served as an internal control to verify equal loading of protein into each electrophoretic well^61^. The intensity of blots was calculated using the ImageJ or the Quantity One^®^ softwares.

### Bioinformatic analysis

The membrane-topology of Nrf1 was predicted using several bioinformatic algorithms, including the TopPred (http://mobyle. pasteur.fr/cgi-bin/portal.py?form=toppred), HeliQuest (http://heliquest.ipmc.cnrs.fr/) and AmphipaSeek (http://npsa-pbil.ibcp. fr/cgi-bin/npsa_automat.pl?page=/NPSA/npsa_amphipaseek.html) programmes. The PEST sequence, as a potential proteolytic cleavage site for proteasome and/or calpain, was found in Nrf1 using the ePESTfind program at http://mobyle.pasteur.fr /cgi-bin/portal.py?#forms::epestfind. The T-Coffee program was employed to align Nrf1 amino acid sequences with those of its orthologues or other known membrane-bound proteins.

### Statistical analysis

The statistical significance of changes in the Nrf1 activity and in the cell viability was determined using the Student’s *t* test or *M*ultiple *An*alysis *o*f *Va*riations (MANOVA). The data are shown as a fold change (mean ± S.D), each of which represents at least 3 independent experiments undertaken on separate occasions that were each performed in either triplicate or quadruplicate.

## Additional Information

**How to cite this article**: Zhang, Y. *et al.* The selective post-translational processing of transcription factor Nrf1 yields distinct isoforms that dictate its ability to differentially regulate gene expression. *Sci. Rep.* **5**, 12983; doi: 10.1038/srep12983 (2015).

## Supplementary Material

Supplementary Information
