# Supplementary material for: The selective post-translational processing of transcription factor Nrf1 yields distinct isoforms that dictate its ability to differentially regulate gene expression
Source: Sci Rep. 2015 Aug 13;5:12983. doi: 10.1038/srep12983 (PMC4534795; doi:10.1038/srep12983)
Supplement: Supplementary Information [file srep12983-s1.pdf]

The selective post-translational processing of transcription factor Nrf1 yields distinct isoforms that dictate its ability to differentially regulate gene expression

Yiguo Zhang[1,2,*], Shaojun Li[1], Yuancai Xiang[1], Lu Qiu[1], Huakan Zhao[1], and John D. Hayes[2]

[1]The Laboratory of Cell Biochemistry and Gene Regulation, College of Medical Bioengineering and Faculty of Life Sciences, University of Chongqing, No. 174 Shazheng Street, Shapingba District, Chongqing 400044, China; [2]Jacqui Wood Cancer Centre, James Arrott Drive, Division of Cancer Research, Medical Research Institute, Ninewells Hospital & Medical School, University of Dundee, DD1 9SY, Scotland, UK. *Correspondence should be addressed to Yiguo Zhang (Email: yiguozhang@cqu.edu.cn; y.z.zhang@dundee.ac.uk)

SUPPLEMENTAL RESULTS

Figure S1

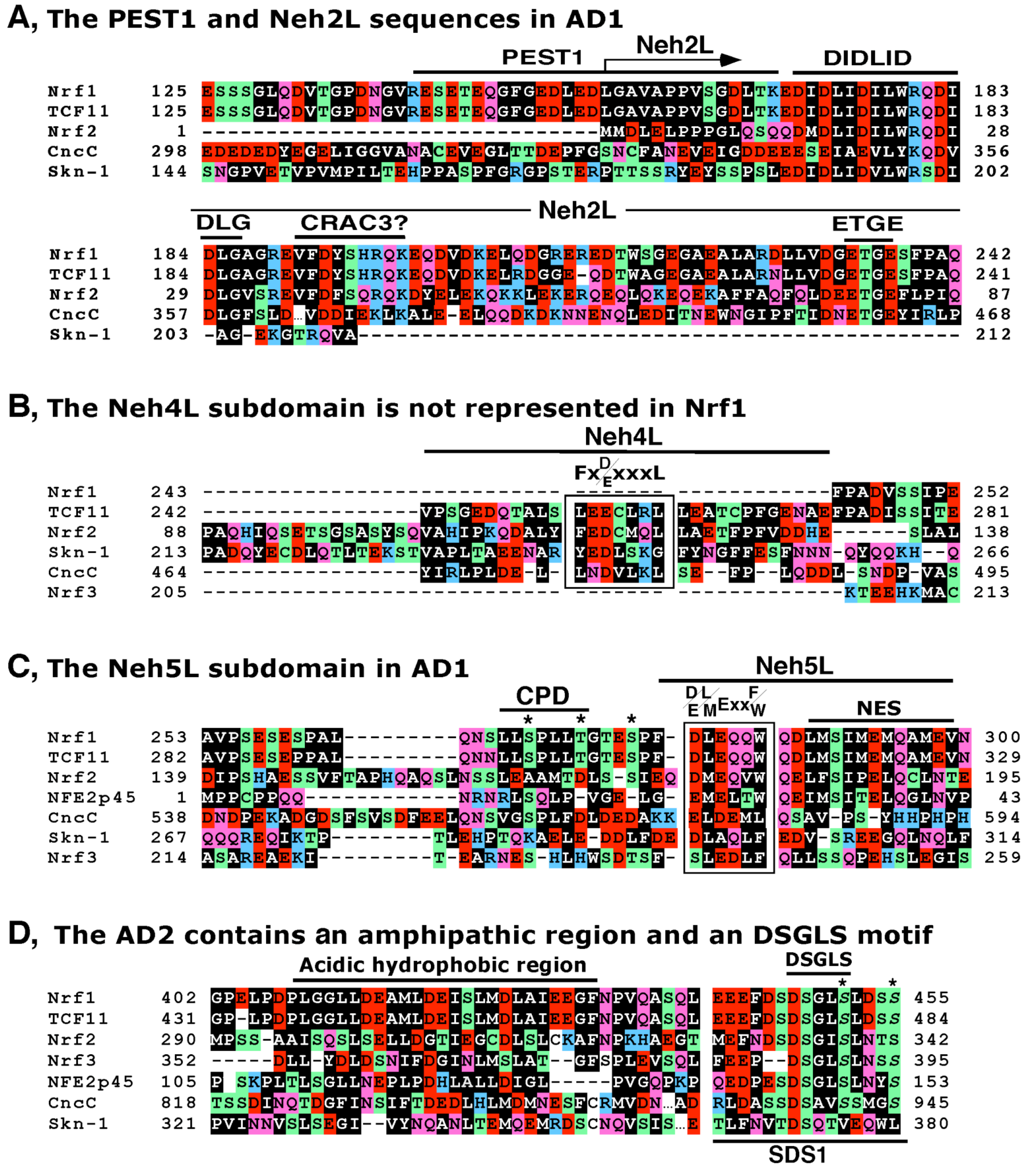

Figure S1. Amino acid alignment of the AD1 and AD2 region from Nrf1 and other CNC family members.
(A) The two potential PEST1 and Neh2L degrons within AD1 of Nrf1. The PEST1 (aa 141-170) sequence overlaps with an N-terminal portion of the Neh2L region (aa 156-242) in AD1 of Nrf1. The PEST1-adjoining DIDLID/DLG element resembles that in Nrf2, Skn-1 and CncC, and thus this region may serve as both a degron and a transactivation element[1]. However, it is notable that the DLG and the ETGE motifs in Nrf1 do not interact with CRL[Keap1] (refs.[2-4]), although they regulate degradation of Nrf2 through the Keap1-scaffolded Cullin-3 ubiquitin ligase-directed proteasome pathway[5, 6]. With the exception of the DIDLID element, most of the Neh2L subdomain, including the ETGE motif, is absent in Skn-1. Importantly, the DIDLID/DLG-flanking CRAC3 motif (aa 191-199) is present only in Nrf1 and its long

TCF11 form, but is not represented in Nrf2 or CncC. This disparity appears to help explain why CRAC3 enables Neh2L in Nrf1 to escape regulation by Keap1, whereas the absence of CRAC motif in Nrf2 and CncC allows them to be regulated by Keap1 (refs.[5, 7]). **(B)** The Neh4L region is absent from the AD1 of Nrf1, but is present in TCF11, Nrf2, Skn-1 and CncC. The Neh4 domain in Nrf2 has been identified as a TAD[8, 9], in which the functional motif F-x-$^{D}/_{E}$-x-x-x-L is boxed to allow comparison with other CNC factors. **(C)** The Neh5L region in AD1 is coupled with the putative Cdc4 phosphodegron (CPD, ^{267}LLSPLLT^{273}). Amongst CNC factors, the Neh5L region is more conserved than Neh2L or Neh4L. The Neh5L region has been shown to act as an essential transactivation element in Nrf1 and Nrf2 (refs.[10, 11]); it contains a functional motif $^{D}/_{E}$-$^{L}/_{M}$-$^{D}/_{E}$-x-x-$^{W}/_{F}$ (boxed) and a nuclear export signal (NES, indicated by horizontal bar). Nrf1-mediated transactivation of target genes by Neh5L is possibly regulated by its adjacent CPD that targets this NHB1-CNC protein to the SCF^{Fbw7}-scaffolded Cullin-1 ubiquitin E3 ligase-proteasome degradation pathway[12]. The star (*) indicates two potential GSK3-recognized phosphorylation sites within the CPD. **(D)** Evolutionary conservation of AD2 amongst different CNC transcription factors. Amino acid alignment of AD2 from distinct CNC proteins demonstrates that this region is highly conserved amongst different species. The AD2 contains an acidic-hydrophobic region that shares certain similarity with DIDLID/DLD and Neh5L regions. Within AD2, its C-terminal SDS1 portion contains a β-TrCP-binding site ^{447}DSGLS^{451}, as described elsewhere[4].

Figure S2

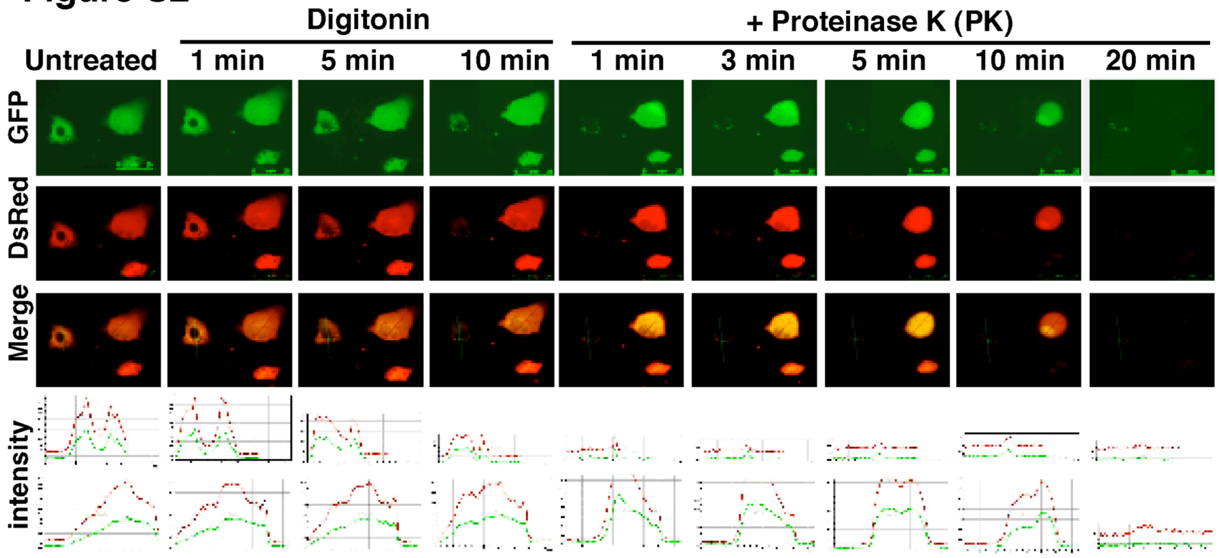

Figure S2. The control imaging of live cells expressing the DsRed-GFP fusion protein.
COS-1 cells expressing the DsRed-GFP fusion protein were subjected to live-cell imaging combined with the *in vivo* membrane protease protection assay. The cells were permeabilized by digitonin (20 μg/ml) for 10 min, before being co-incubated with PK (50 μg/ml) for 20 min. In the time course, real-time images were acquired using the Leica DMI-6000 microscopy system. The merged images of Nrf1/GFP with ER/DsRed are placed (on *the third row of panels*), whereas changes in the intensity of their signals are shown graphically (*bottom*). The features of arrow-indicated cells are described in the main text. Overall, the images shown herein are a representative of at least three independent experiments undertaken on separate occasions that were each performed in triplicate (n=9). In determining dynamic repartitioning of Nrf1 to dislocate out of the ER by live imaging of COS-1 cells co-expressing either Nrf1/GFP or N275/GFP with DsRed (Figs. 1 & 2), the control imaging of the DsRed-GFP fusion protein was shown herein. The double fluorescent intensity of DsRed/GFP that was principally located in the cytoplasm was decreased by largely 25% or 50% after incubation of the *left* cells with digitonin for 5 or 10 min, respectively, when compared with the intensity determined from untreated conditions (*the second lower graphs*). The remaining signals of DsRed/GFP were suddenly abrogated by 1-min incubation with and almost completely destroyed with 3 min by the protease. This demonstrates that a cytoplasmic fraction of DsRed-GFP is not protected by membranes against PK digestion. In addition, an exception was made in the case that relatively over-expressed DsRed-GFP (*the right upper cells*) appeared to be unaffected by digitonin, and the fluorescent signals seemed to be shortly enhanced within 3 min of incubation with PK because the cell was shrunk to approximately 30% of its original size under untreated conditions (*the bottom graphs*). Indeed, the over-expressed DsRed-GFP was also obviously digested by PK incubation for 10 min, and then gradually disappeared by PK within 15 min. By comparison with the cytoplasmic fraction of DsRed-GFP, the nuclear fraction of the fusion protein may be partially protected by the nuclear membranes such that it is not sensitive to PK.

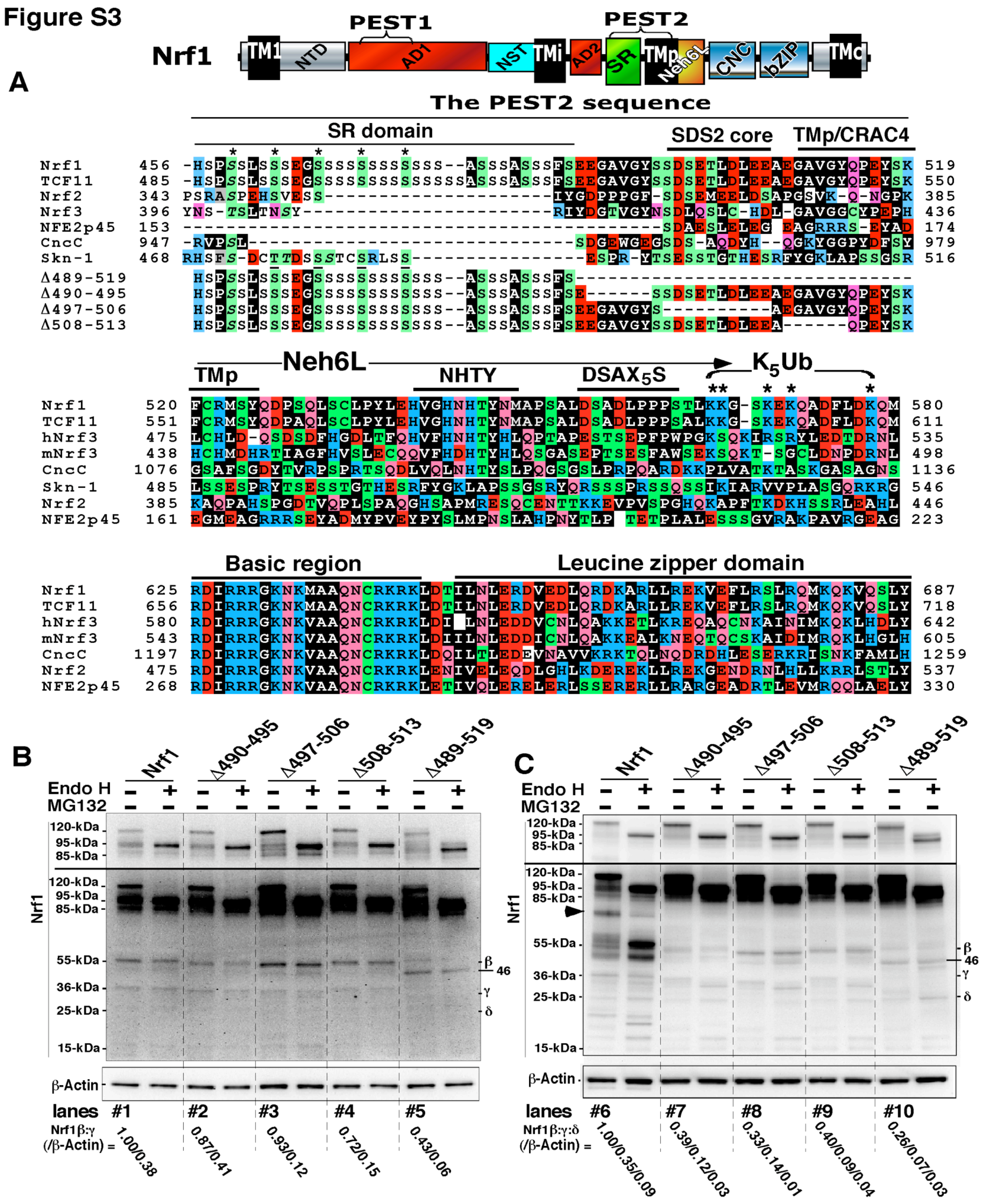

Figure S3. The protealytic processing of Nrf1 is differentially regulated by the PEST2 and SDS2-adjoining sequences. **(A)** The putative PEST2 sequence is composed of the SR domain and the SDS2 region overlapping the Neh6L domain. The PEST2 sequence contains the serine-repeat (SR) domain and the N-terminal one-third of Neh6L. The alignment of PEST2 amino acid sequences from different CNC transcription factors indicates that SR is specific for Nrf1 but is not present in the other homologous proteins. However, the core SDS2-adjcent sequence is represented in all CNC factors, but the CRAC4 (^{511}VxYxxxK^{519}) motif appears to be specific for Nrf1. Conserved GSK-3 phophorylation sites are situated in the N-terminal border of the SR region. Like AD2, the SR region contributes to stimulation of Nrf1-mediated transactivation of target genes by glucose deprivation, whilst PEST2 and SDS2 contribute to the negative regulation of Nrf1. *The lower two panels* show an alignment of amino acids covering the net positive regions of Nrf1, revealing that the Neh6L domain is less conserved than the bZIP domain amongst the family members. In Nrf1, the net positive portion of Neh6L comprises NHTY (aa 540-548) and DSAX$_5$S (aa 554-562), immediately followed by a lysine-rich cluster (K$_5$-UB, aa 565-578, that has potential to be ubiquitylated by SCF$^{\beta\text{-TrCP}}$). Both Cys^{521} and Cys^{533} are located within and around the TMp sequence, and possibly respond to redox stress. **(B** and **C)** Internal deletion of SDS2 or most of CRAC4 had different effects on the abundance of 36-kDa Nrf1γ and/or 55-kDa Nrf1β with distinct activities. The total lysates of cells expressing Nrf1, or its mutants (lacking SDS2 and its adjacent peptides), were subjected to deglycosylation reactions with Endo H, without treatment with the proteasomal inhibitor MG132, followed by western blotting with the V5 antibody. The resultant immunoblot were obtained from two different experimental settings of COS-1 cells with distinct confluences of 50% or 70%, that had been transfected for 6 or 12 h before being recovered for 18 h in fresh complete media, respectively. The upper and middle images were herein presented for different times of exposure to the supersensitive reagent. Subsequently, the intensity of the 55-, 36- or 25-kDa protein blots (presented *in the middle panel*) was estimated by dividing the value for all short Nrf1 isoforms with that for β-actin, and then the relative amount of these short isoforms (detected on the same gel) was normalized to the basal level (designated 1.0) of the 55-kDa protein measured from untreated cells expressing wild-type Nrf1. The resultant ratios of Nrf1β, Nrf1γ and Nrf1δ were shown in the bottom.

Figure S4

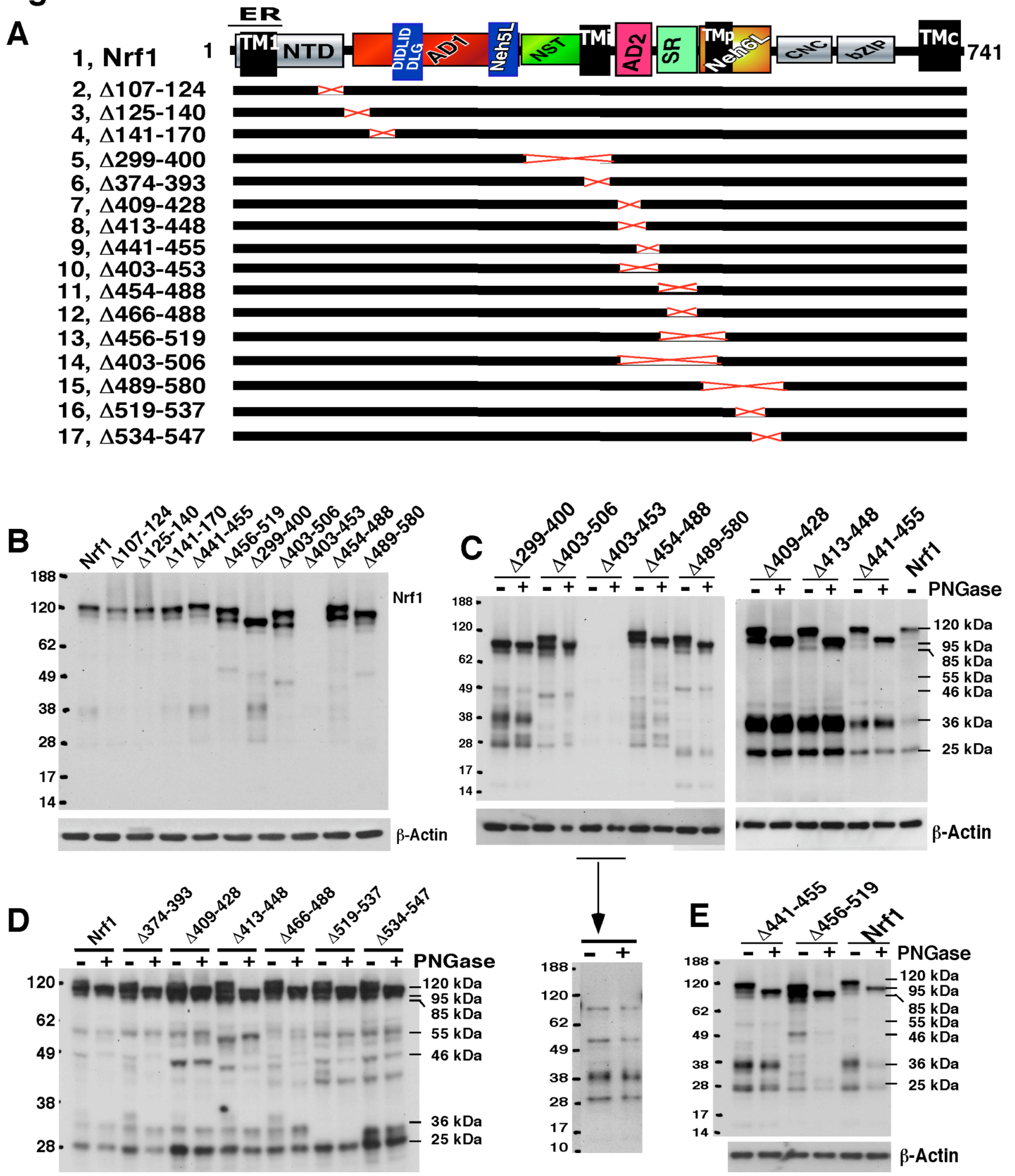

Figure S4. Regulation of Nrf1 proteolysis and glycosylation by AD2 and its adjoining regions

(A) Schematic diagram illustrating distinct regions of Nrf1 and its mutagenesis mapping. The *upper* cartoon shows nine domains of Nrf1, with some conserved regions being indicated. The *lower* diagrammatic shows a series of internal mutants lacking distinct regions of Nrf1, that are indicated by cross marks on the white background. **(B)** Differential expression of 95-kDa and 120-kDa Nrf1 proteins. Total lysates from cells that contained expression vectors for Nrf1 or its mutants were resolved by 4-12% LDS/NuPAGE and analysed by western blotting. The $Nrf1^{\Delta 404\text{-}453}$ mutant, which lacks AD2, is an unstable protein. By contrast, two major proteins of 120-kDa and 95-kDa were clearly observed in cells expressing the $Nrf1^{\Delta 456\text{-}519}$ mutant (lacking the PEST2 sequence), the $Nrf1^{\Delta 403\text{-}506}$ mutant (lacking AD2 and most of PEST2), or $Nrf1^{\Delta 454\text{-}488}$ (lacking the SR domain). Forced expression of the $Nrf1^{\Delta 299\text{-}400}$ mutant (lacking the NST glycodomain) gave rise to a non-glycosylated 95-kDa protein, whereas expression of the $Nrf1^{\Delta 489\text{-}580}$ mutant (lacking the Neh6L domain) gave rise to a glycoprotein of 110 kDa, with smaller minor proteins. **(C)** AD2 and its adjacent NST domain influence the proteolytic processing of Nrf1 to yield its 25-kDa and 36-kDa forms. Cells expressing Nrf1 or its mutants were harvested in a glycoprotein-denaturing buffer. The cell lysates were (+), or were not (-), incubated for 1 h with 500 units of PNGase F, before they were resolved using 4-12% LDS/NuPAGE, in Tris-Bis running buffer, and immunoblots probed with V5 antibodies. The amount of protein applied to each polyacrylamide gel sample well was adjusted to ensure equal loading of β-gal activity. The position of the Nrf1 isoforms was estimated to be 120, 95, 85, 55, 46, 36 and 25 kDa. In addition, β-actin served as an internal control of protein loading. The $Nrf1^{\Delta 403\text{-}453}$ protein appears to be unstable and non-glycosylated (*lower*). The degraded polypeptide ladders from in the $Nrf1^{\Delta 403\text{-}506}$, $Nrf1^{\Delta 454\text{-}488}$ and $Nrf1^{\Delta 489\text{-}580}$ mutant proteins appear to be relatively faint and poorly resolved. **(D)** Formation of the processed 36-kDa Nrf1γ isoform was prevented by loss of residues 519-537 (lacking a TMp-flanking portion of Neh6L). Deglycosylation of cell lysates with PNGase F, followed by western blotting, was carried out as above. The β-gal activity was measured and the amount of protein applied to each polyacrylamide sample well was adjusted to ensure equal loading of β-gal activity. **(E)** Formation of the processed 25-kDa Nrf1δ and 36-kDa Nrf1γ isoforms was inhibited by loss of residues 456-519 (that cover the PEST2 sequence containing SR and TMp-flanking SDS2 peptide). Cell sampling, deglycosylation and western blotting were performed as above.

Figure S5

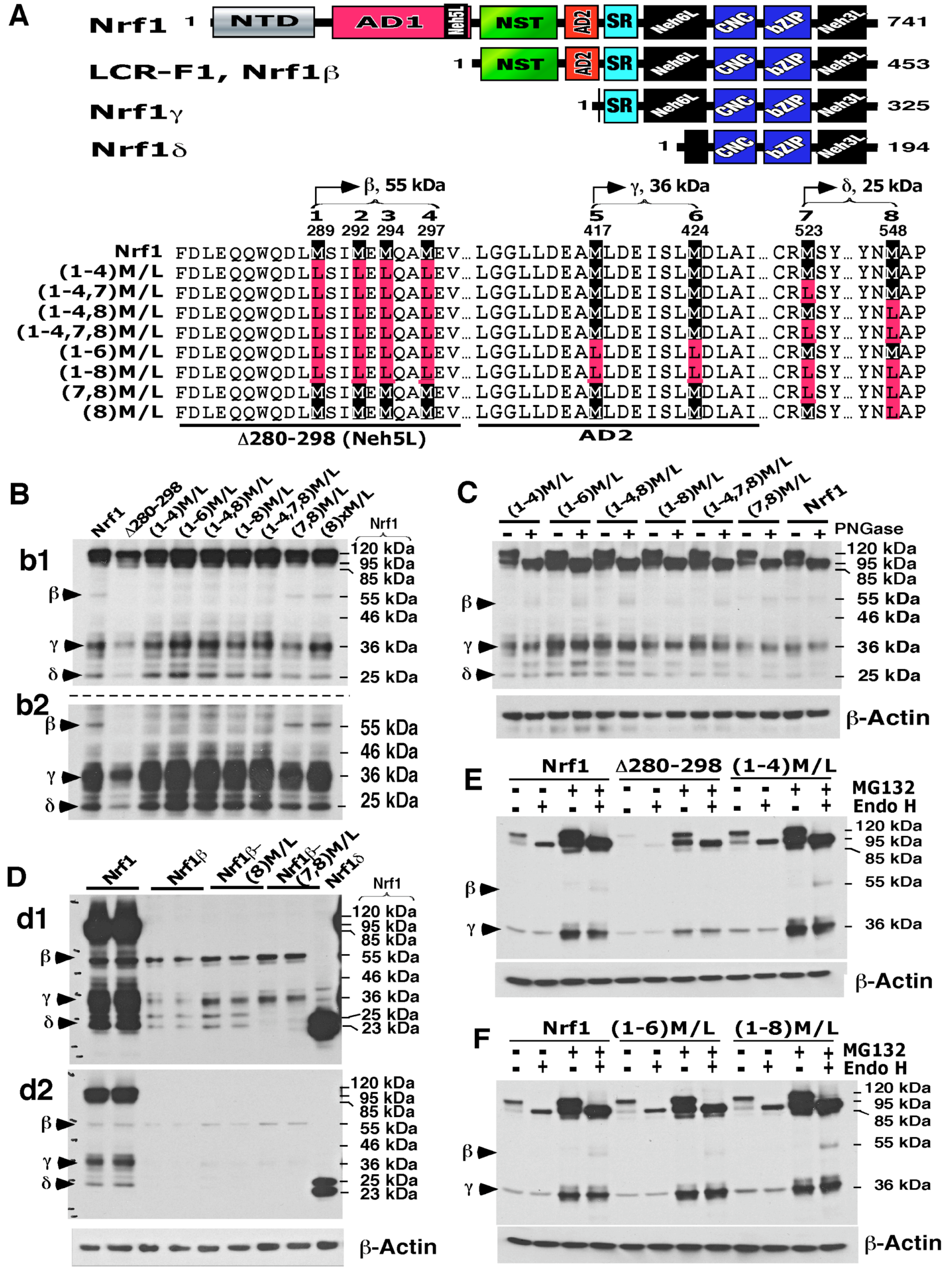

Figure S5. Nrf1β, Nrf1γ and Nrf1δ arise through translation from internal ATG start codons

(A) Schematic representation of Nrf1, Nrf1β, Nrf1γ and Nrf1δ with distinct Met-to-Leu mutations**.** Besides proteolysis, i) the 55-kDa LCR-F1/Nrf1β (aa 292-741) isoform can also arise from in-frame translation through internal start codons at Met[289], Met[292], Met[294], or Met[297] (refs.[13-15]); ii) the 36-kDa Nrf1γ (aa 417-741) isoform is likely produced by the internal translation initial signal at Met[417] or Met[424]; iii) the 25-kDa Nrf1δ (aa 548-741) isoform is predicted to arise from translation starting at Met[523] or Met[548], as described elsewhere[16, 17]. To test whether internal translational initiation signal accounts for the 55-kDa, 36-kDa and 25-kDa isoforms, we created a series of Met-to-Leu mutants by PCR-directed mutagenesis. **(B)** The formation of 55-kDa Nrf1β is prevented by mutation of all ATG condons for Met[289], Met[292], Met[294], and Met[297]. Cell lysates of cells expressing Nrf1 or its Met-to-Leu Mutants were examined by western blotting and revealed little 55-kDa protein. The whole gel was exposed to X-ray film for various times, part of which was cropped as shown (*bottom*). **(C)** Production of 55-kDa Nrf1β by proteolysis during deglycosylation**.** Cell lysates expressing Nrf1 or its Met-to-Leu Mutants were subjected to deglycosylation by PNGase F, followed by western blotting. **(D)** The formation of 25-kDa Nrf1δ rather than 36-kDa Nrf1γ is prevented by mutation of both

Met523 and Met548. Expression of Nrf1, or Nrf1β (aa 292-741) and its mutants, as well as Nrf1δ (aa 548-741), was visualized by western blotting. The whole gel was exposed to X-ray film for 30 sec (*upper*) or 5 sec (*middle*). In addition, it is plausible that 55-kDa Nrf1β is proteolytically processed to yield 36-kDa Nrf1γ and 25-kDa Nrf1δ. **(E** and **F)** The abundance of 120, 95, 85, 55 and 36-kDa Nrf1 forms is increased by treatment with MG132, but the abundance of the Met-to-Leu Nrf1 mutants is not increased by this inhibitor. After recovery from transfection, the cells were treated with 5 μmol/L MG132 for 2 h before being harvested in a glycoprotein-denaturing buffer; the control was incubated in buffer alone. Thereafter, the cell lysates were subjected to deglycosylation with Endo H, and then resolved using 4-12% LDS/NuPAGE, in Tris-Bis running buffer, before immunoblotting. Lastly, the position of electrophoretic migration of the Nrf1 isoforms was estimated to be 120, 95, 85, 55, 46, 36 and 25 kDa, and β-actin was employed as an internal control.

Figure S6

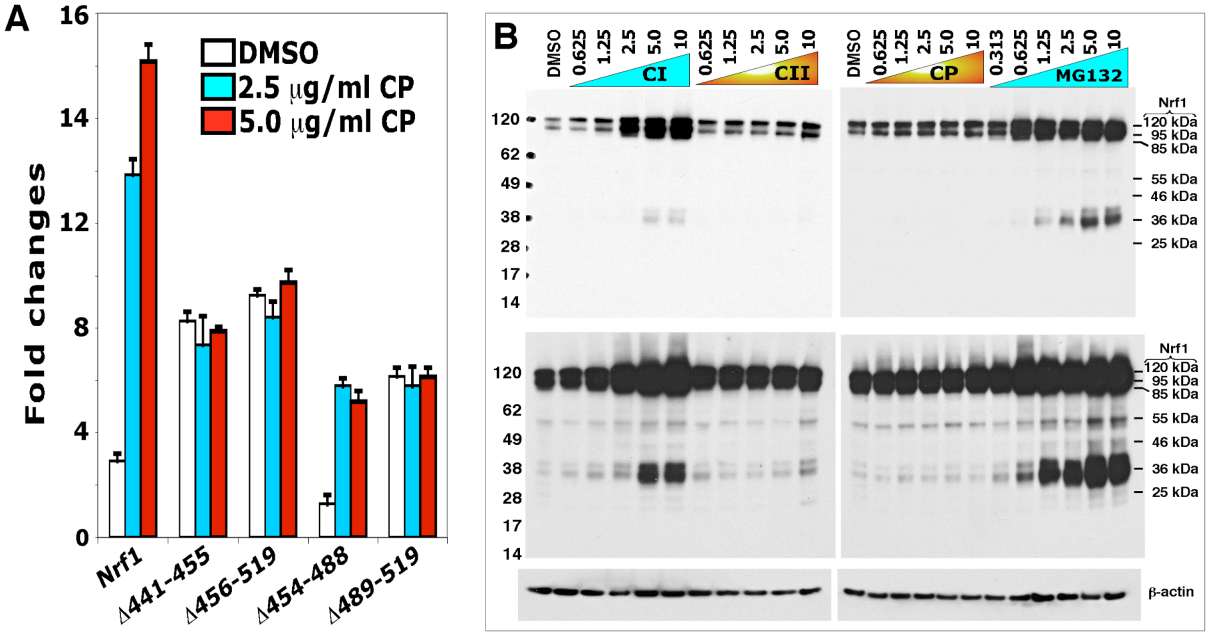

Figure S6. Both proteasome and calpain inhibitors have dual opposing effects on the processing of Nrf1.
(A) Calpeptin does not increase transactivation by Nrf1 mutants that decrease the abundance of Nrf1γ. COS-1 cells that had been transfected with an expression construct for Nrf1 or its mutants, along with an ARE-driven reporter plasmid, were treated overnight with 2.5 μg/ml calpeptin (CP) or 5.0 μg/ml CP in a 5.5-mM glucose medium. Then, luciferase activity in total lysates was measured by standard methods. **(B)** Distinct effects of proteasome and calpain inhibitors on Nrf1 protein pattern**.** Cells expressing Nrf1 were recovered for 8 h in the 25-mM glucose medium and were then transferred to the fresh 5.5-mM glucose medium for an additional 18 h. Thereafter, they were treated with different doses of inhibitors for 3 h, before being harvested in the denaturing buffer. Subsequently, these lysates were resolved using LDS/NuPAGE containing 4-12% Tris-Bis gel, followed by western blotting with V5 antibodies. X-ray films were exposed to the immunoblot for 5 sec (*upper*) or 30 sec (*middle*).

Figure S7. Opposing regulation of Nrf1-targeted luciferase reporter gene by p97/VCP and Hrd1.
HEK293 cells (3 × 105) were seeded in each of 6-well plates and cultured overnight. The cells were co-transfected for 8 h by Liofectamine 200 containing 1 μg of Nrf1/pcDNA3.1/V5HisB, 1 μg of *GSTA2*-6×ARE-Luc and 0.3 μg of pRL-TK, together with 50 nmol/L of each of p97siRNA, Hrd1siRNA and negative control siRNA. The transfected cells were then allowed to recover for 24 h in complete DMEM containing 10% FBS and 25 mM glucose. The cell lysates were subjected to real-time PCR to examine the expression of p97 (**A**, *left panel*) and Hrd1 (**B**, *left panel*), followed by reporter gene activity assays (*right panels*). The data were calculated as a fold change (mean ± S.D) of Nrf1 transactivation activity. Significant increases ($, $p<0.05$ and $$, $p<0.001$, n=8) and decreases (*$p<0.05$, **$p<0.001$, n=8) are indicated.

Figure S7

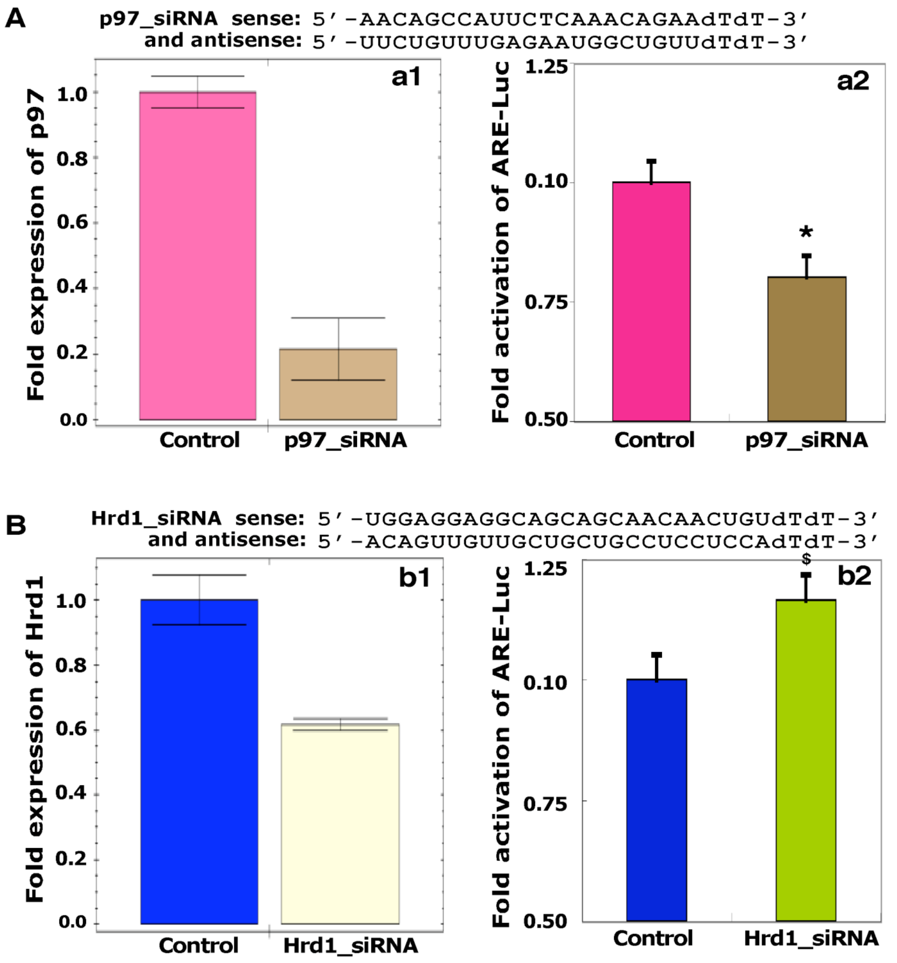

SUPPLEMENTAL REFERENCES

1. Walker, A. K. et al. A conserved transcription motif suggesting functional parallels between Caenorhabditis elegans SKN-1 and Cap'n'Collar-related basic leucine zipper proteins. *J. Biol. Chem.* **275**, 22166-22171 (2000).
2. Zhang, Y., Crouch, D. H., Yamamoto, M. & Hayes, J. D. Negative regulation of the Nrf1 transcription factor by its N-terminal domain is independent of Keap1: Nrf1, but not Nrf2, is targeted to the endoplasmic reticulum. *Biochem. J.* **399**, 373-385 (2006).
3. Zhao, R. et al. Long isoforms of NRF1 contribute to arsenic-induced antioxidant response in human keratinocytes. *Environ. Health Perspect.* **119**, 56-62 (2011).
4. Tsuchiya, Y. et al. Dual Regulation of the Transcriptional Activity of Nrf1 by beta-TrCP- and Hrd1-Dependent Degradation Mechanisms. *Mol. Cell Biol.* **31**, 4500-4512 (2011).
5. Padmanabhan, B. et al. Structural basis for defects of keap1 activity provoked by its point mutations in lung cancer. *Mol. Cell* **21**, 689-700 (2006).
6. McMahon, M., Thomas, N., Itoh, K., Yamamoto, M. & Hayes, J. D. Dimerization of substrate adaptors can facilitate cullin-mediated ubiquitylation of proteins by a "tethering" mechanism: a two-site interaction model for the Nrf2-Keap1 complex. *J. Biol. Chem.* **281**, 24756-24768 (2006).
7. Sykiotis, G. P. & Bohmann, D. Keap1/Nrf2 signaling regulates oxidative stress tolerance and lifespan in Drosophila. *Dev. Cell* **14**, 76-85 (2008).
8. Itoh, K. et al. Keap1 represses nuclear activation of antioxidant responsive elements by Nrf2 through binding to the amino-terminal Neh2 domain. *Genes Dev.* **13**, 76-86 (1999).
9. Katoh, Y. et al. Two domains of Nrf2 cooperatively bind CBP, a CREB binding protein, and synergistically activate transcription. *Genes Cells* **6**, 857-868 (2001).
10. Zhang, Y., Lucocq, J. M. & Hayes, J. D. The Nrf1 CNC/bZIP protein is a nuclear envelope-bound transcription factor that is activated by t-butyl hydroquinone but not by endoplasmic reticulum stressors. *Biochem. J.* **418**, 293-310 (2009).
11. Zhang, J. et al. Nrf2 Neh5 domain is differentially utilized in the transactivation of cytoprotective genes. *Biochem. J.* **404**, 459-66 (2007).
12. Biswas, M., Phan, D., Watanabe, M. & Chan, J. Y. The Fbw7 tumor suppressor regulates nuclear factor E2 related factor 1 (Nrf1) transcription factor turnover through proteasome-mediated proteolysis. *J. Biol. Chem.* **286**, 39282-39289 (2011).
13. Caterina, J. J., Donze, D., Sun, C. W., Ciavatta, D. J. & Townes, T. M. Cloning and functional characterization of LCR-F1: a bZIP transcription factor that activates erythroid-specific, human globin gene expression. *Nucleic Acids Res.* **22**, 2383-2391 (1994).
14. Husberg, C., Murphy, P., Martin, E. & Kolsto, A. B. Two domains of the human bZIP transcription factor TCF11 are necessary for transactivation. *J. Biol. Chem.* **276**, 17641-17652 (2001).
15. Johnsen, O. et al. Small Maf proteins interact with the human transcription factor TCF11/Nrf1/LCR-F1. *Nucleic Acids Res.* **24**, 4289-4297 (1996).
16. Zhang, Y. Molecular and cellular control of the Nrf1transcription factor: An integral membrane glycoprotein. *Vdm Verlag Dr. Müller Publishing House Germany*. **The first edition**, pp1-264 (2009).
17. Novotny, V., Prieschl, E. E., Csonga, R., Fabjani, G. & Baumruker, T. Nrf1 in a complex with FosB, c-Jun, JunD and ATF2 forms the AP1 component at the TNFα promoter in stimulated mast cells. *Nucleic Acids Res.* **26**, 5480-5485 (1998).
